# Mis-expression of *grainyhead-like* transcription factors in zebrafish leads to defects in enveloping layer (EVL) integrity, cellular morphogenesis and axial extension

**DOI:** 10.1038/s41598-017-17898-7

**Published:** 2017-12-14

**Authors:** Lee B. Miles, Charbel Darido, Jan Kaslin, Joan K. Heath, Stephen M. Jane, Sebastian Dworkin

**Affiliations:** 1 Department of Physiology, Anatomy and Microbiology, La Trobe University, Bundoora, VIC 3086 Australia; 2 The Victorian Comprehensive Cancer Centre, Peter MacCallum Cancer Centre, Parkville, VIC 3050 Australia; 3 The Australian Regenerative Medicine Institute, Monash University, Clayton, VIC 3168 Australia; 4 Department of Chemical Biology, The Walter and Eliza Hall Institute, Parkville, VIC, 3050 Australia; 5 Department of Medicine, Monash University Central Clinical School, Prahran, VIC 3181 Australia; 6 Department of Hematology, Alfred Hospital, Prahran, VIC 3181 Australia

## Abstract

The *grainyhead-like* (*grhl*) transcription factors play crucial roles in craniofacial development, epithelial morphogenesis, neural tube closure, and dorso-ventral patterning. By utilising the zebrafish to differentially regulate expression of family members *grhl2b* and *grhl3*, we show that both genes regulate epithelial migration, particularly convergence-extension (CE) type movements, during embryogenesis. Genetic deletion of *grhl3* via CRISPR/Cas9 results in failure to complete epiboly and pre-gastrulation embryonic rupture, whereas morpholino (MO)-mediated knockdown of *grhl3* signalling leads to aberrant neural tube morphogenesis at the midbrain-hindbrain boundary (MHB), a phenotype likely due to a compromised overlying enveloping layer (EVL). Further disruptions of *grhl3*-dependent pathways (through co-knockdown of *grhl3* with target genes *spec1* and *arhgef19*) confirm significant MHB morphogenesis and neural tube closure defects. Concomitant MO-mediated disruption of both *grhl2b* and *grhl3* results in further extensive CE-like defects in body patterning, notochord and somite morphogenesis. Interestingly, over-expression of either *grhl2b* or *grhl3* also leads to numerous phenotypes consistent with disrupted cellular migration during gastrulation, including embryo dorsalisation, axial duplication and impaired neural tube migration leading to cyclopia. Taken together, our study ascribes novel roles to the *Grhl* family in the context of embryonic development and morphogenesis.

## Introduction

The *grainyhead-like* (*Grhl*) gene family, vertebrate orthologues of the antecedent Drosophila gene *grainyhead* (*grh*), are a highly conserved family of developmentally-critical transcription factors. Comprising three members in vertebrates (*Grhl1-3*), with a further sub-functionalisation of *grhl2* in zebrafish (*grhl2a* and *grhl2b* respectively), this family regulates diverse processes which ensure correct cell and tissue patterning and development, including formation of the *Drosophila* cuticle^1^ and skin barrier^2,3^, polarity-dependent processes of epidermal migration and wound healing^4,5^, neuroblast proliferation and apoptosis^6–8^, convergence-extension (CE) mediated movements in neural tube closure and axial patterning^9,10^, and development of the craniofacial skeleton and palatal fusion^11–14^ in vertebrates. Importantly, almost all phenotypes described in *Drosophila* resulting from loss of *grh* have thus far also been characterised in vertebrate models, further highlighting the remarkable conservation of gene function over 700 million years of evolution^2^.

The three vertebrate orthologues vary in their functional importance during development, although all are predominantly expressed in epithelia^15^. *Grhl1* is implicated in the development of both auditory^16^ and epidermal^17–19^ tissues, although *Grhl1* ^*−/−*^ mice are viable and fertile^18^. Conversely, loss of either *Grhl2* or *Grhl3* in mice leads to severe defects in cell survival, migration, integration, fusion and patterning. Although the early embryonic lethality (between embryonic day [E] 9.5-E11.5 of development) of *Grhl2* knockout animals has precluded analyses of skin barrier formation, craniofacial patterning and wound healing, *Grhl2* ^*−/−*^ mutants present with severe exencephaly, fully-penetrant split-face and maxillary clefting, an open neural tube and defects in brain cellularity and neuronal survival^8,10,20^. Similarly, loss of *Grhl3* leads to fully penetrant thoracolumbosacral spina bifida, occasional exencephaly, neurocognitive and behavioural disorders, defects in epithelial development of the bladder and intestine and convergence-extension defects^9,21–23^; although embryos survive to birth, *Grhl3* ^*−/−*^ embryos die soon after due to rapid trans-epidermal water loss as a consequence of an impaired skin barrier and dehydration following exposure to a terrestrial environment^24^.

Although numerous studies have determined key developmental roles following loss of *Grhl* expression, the over-expression of *Grhl* factors has been difficult to model, largely limited to the generation of transgenic mice. Whereas transgenic expression of *Grhl3* has been reported^25^, utilising a BAC to restore *Grhl3* expression and rescue spina bifida-like defects in a hypomorphic *Grhl3* model (the *curly-tail* mouse), this study did not report any over-expression-related phenotypic abnormalities in these mice, nor were over-expression studies performed using the BAC construct in WT mice. However, over-expression of *Grhl2* (in the spontaneously occurring axial defects [*axd*] mouse) was reported to lead to an increase in cellular proliferation and neural tube defects^26,27^. These data suggest that tight homeostatic regulation of both *Grhl2* and *Grhl3* is required to ensure correct neural tube morphogenesis and axial development, although differences in gene function are likely to exist between disparate animal models.

Although virtually all *grh*-dependent phenotypes in *Drosophila* are conserved in vertebrates, the full spectrum of vertebrate-specific developmental processes in embryogenesis remains incompletely characterised. Utilising the highly genetically tractable zebrafish model, we have previously shown that loss of *grhl2b* (the fish orthologue most closely related to *Grhl2*) leads to aberrant midbrain-hindbrain boundary (MHB) morphogenesis and patterning^28^ whereas loss of *grhl3* leads to impaired craniofacial cartilage growth and development^12^. Here, we utilised the zebrafish model to further uncover novel phenotypes following genetic deletion of *grhl3*, and knockdown and over-expression of zebrafish and mammalian *grhl2b*, *Grhl2* and *Grhl3*, and ascribe novel cellular and morphogenetic functions regulated by this transcription factor family in vertebrates.

## Methods

### Zebrafish microinjection techniques

All animal experimentation was conducted under ethical approval granted by the Monash University, La Trobe University and Walter and Eliza Hall Animal Ethics Committees (AEC), and performed in accordance with all relevant guidelines and regulations. ATG-blocking morpholinos (MO; Gene-Tools) were designed to inhibit translation of *arhgef19* (GAGAGATTCCATAGCCAGGAAGCAT) and *spec1* (CAATCTTATGCCAGAACGCACTCAT), and these were injected at sub-phenotypic dosages with MOs targeting either *grhl2b* or *grhl3* as reported by us previously^12,28^. Full-length cDNA isoforms of murine *Grhl2* and *Grhl3*, and zebrafish *grhl2b* and *grhl3* were cloned by PCR, inserted into the expression vector pCS2 + , and verified by sequencing. mRNA for injection was generated using the mMESSAGE mMACHINE Sp6 transcription kit (Ambion) and precipitated using Ethanol/LiCl. All *in-situ* hybridisation and imaging techniques were as reported previously^28,29^.

### Validation of *grhl2b* and *grhl3* morpholino specificity

Specific anti-sense oligonucleotides targeting both *grhl2b* and *grhl3* used in the present study have been rigorously validated and reported by us previously. Pertaining to MOs used to inhibit *grhl2b*, we utilised both ATG- and splice-blocking MOs to show reproducibility of loss-of-*grhl2b* phenotype in the brain and otic vesicle, loss of midbrain-hindbrain boundary markers (*eng2a*, *wnt1*, *pax2a* and *her5*), confirmed presence of non-spliced *grhl2b*-transcript using the splice-blocking MO, and confirmed functional rescue of phenotypes using zebrafish *grhl2b* mRNA (with silent mutations introduced at the ATG site to prevent inhibition by the ATG-MO), murine *Grhl2* mRNA, and target gene (*eng2a*) mRNA^28^. Pertaining to MOs used to inhibit *grhl3*, we again utilised both ATG- and splice-blocking MOs to show reproducibility of loss-of-*grhl3* phenotype in the craniofacial skeleton, loss of neural crest and pharyngeal markers (*edn1*, *dlx3*, *hand2*), confirmed presence of non-spliced *grhl3*-transcript using the splice-blocking MO, and confirmed functional rescue using both murine *Grhl3* mRNA and target gene (e*dn1*) mRNA^12^. The negative 5-base pair mismatch *grhl3* control morpholino sequence was TGAcAGgCTCAATgTCCTTcGTgAT.

### Generation of *grhl3*^*−/−*^ fish via CRISPR/Cas9 mediated deletion

The target site for generation of *grhl3* single guide (sg) RNAse was predicted using ZiFit (http://zifit.partners.org/ZiFiT/). Oligonucleotides (IDT) encoding the determined target site (GGTTGCCATGATTTCTGCGA) were cloned into the plasmid pDR274^30^, and sequence-verified plasmids were used as a template to transcribe sgRNA using the MEGAscript T7 kit (Ambion) as per manufactures recommendations. sgRNA, Cas9 nuclease (NEB M0386S), and a specific targeted STOP codon cassette^31^ (CTCTCCTGCCTCAACCGTCGGTCATGGCGTTTAAACCTTAATTAAGCTGTTGTAGCAGAAATCATGGCAATCGGA) were co-injected into the cell of single-cell stage WT Tuebingen zebrafish embryos. sgRNA was injected at concentrations ranging between 20–750 pg/nl, with greater efficiency of cutting observed at higher, as previously reported for other sgRNA^32^, and Cas9 protein was injected at a final concentration of 320 ng/μl. DNA sequencing using specific oligonucleotides flanking the target site (F: CAGCTCTTCCCCTGAACTTG, R: ACATAAATGCGGACCTCAGGTGT) were used to determine genomic changes in injected embryos. DNA was isolated from injected embryos in 20 μl 50 mM NaOH, and heated to 95 °C for 10 min. The solution was neutralised by adding 2 μl 1 M Tris-HCL, pH 8.0. Samples were pulse centrifuged to pellet debris, and supernatant used as a template for subsequent PCR reactions. Presumed nonsense-mediated decay was detected by firstly isolating mRNA from 50 embryos of each of control and *grhl3* ^*−/−*(+*14bp*)^ mutant embryos at 80% epiboly; cDNA was synthesised using SuperscriptIII (Invitrogen) according to manufacturer’s instructions. One μl of cDNA was used to confirm comparable expression of β*-actin* by Q-RT-PCR, as well as expression of *grhl3* transcript by standard PCR, using a forward primer (CACATATTTAACCACAAGCACACC) in Exon 1, and a reverse primer (AATGCTCGATGATGTTGTCG) in Exon 15 to determine relative abundance of *grhl3* transcript.

### Confocal Microscopy

Embryos were fixed in 2% Paraformaldehyde (PFA) overnight and mounted in 2% Low melting temperature agarose. Samples were stained for F-actin before imaging with Rhodamine-Phalloidin (Life Technologies) as per manufacturers’ recommendations. Prior to imaging, embryo heads were dissected from the body and embedded in 4% low melting point agarose under a coverslip. Serial confocal images were taken on a Nikon C1 confocal microscope with either a 20x or 40x lens. Figure preparation was performed using Illustrator CS5 (Adobe).

### Cell area and number measurements

The area of EVL cells over the midbrain-hindbrain boundary was determined using automated quantitation and imaging software (FiJi) to manually outline all individual cells within a defined region of 2500 μm^2^, derived from confocal image stacks. Cell counts were also determined manually for all cells within the defined 2500 μm^2^ region.

### Statistics

Quantitative data derived from at least three independent experiments; descriptive statistics are mean ± SEM of data for (n) individuals or (n) independent experiments. GraphPad prism was used for unpaired 2-tailed Student’s T-tests, with normally-distributed continuous variables. P values are as follows * = P ≤ 0.05, ** = P ≤ 0.01, *** = P ≤ 0.001.

## Results

### *grhl3* deletion leads to severe epithelial disruption and embryo rupture prior to gastrulation

**Figure 1:**
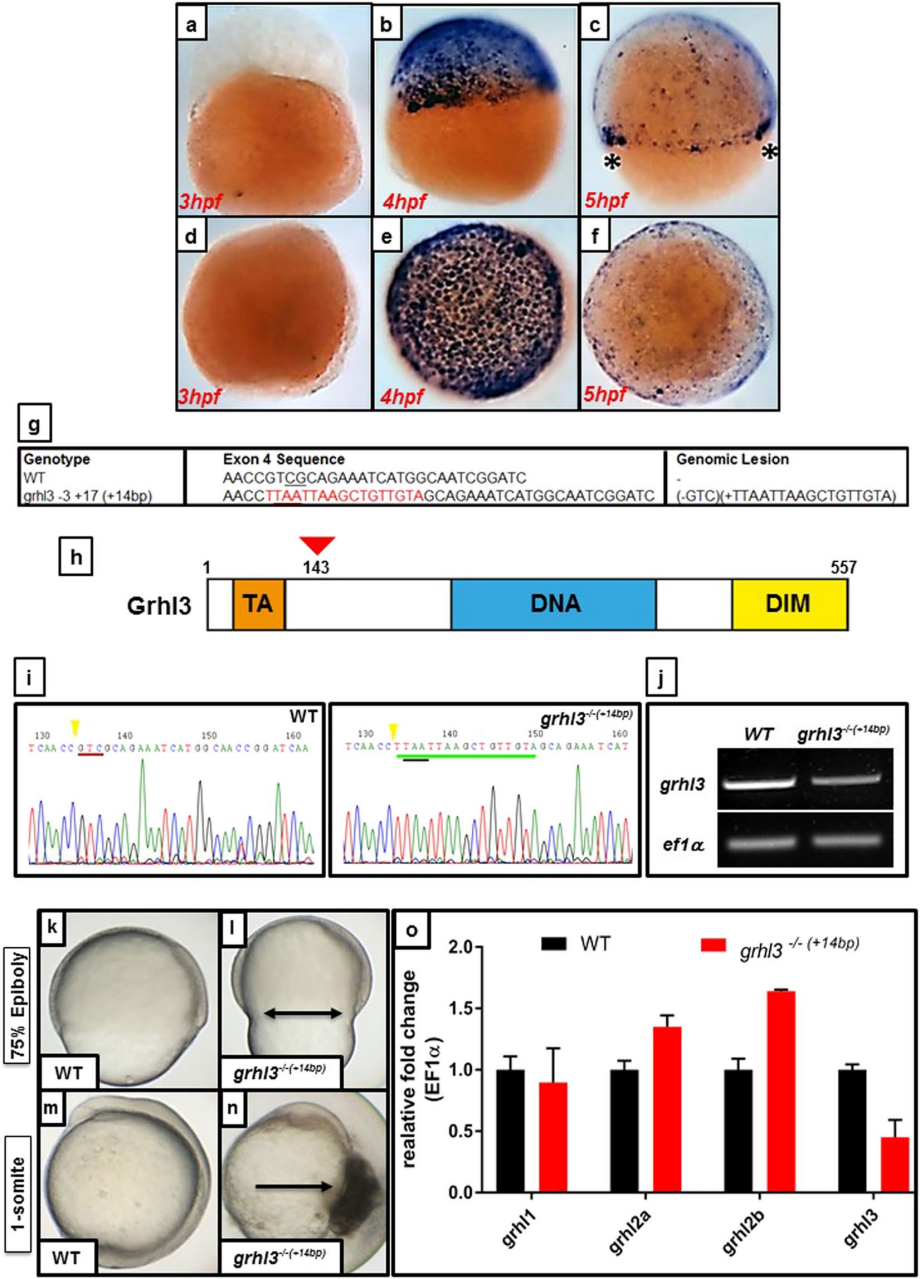
CRISPR-Cas9 mediated generation of a *grhl3* deletion zebrafish model. (**a**–**f**) ISH expression of *grhl3* at 3–5 hpf, shown in lateral (**a**–**c**) and dorsal (**d**–**f**) views. *grhl3* is not maternally deposited (**a**,**d**), but is strongly and specifically expressed within all EVL cells at ~4 hpf, corresponding temporally to the maternal-zygotic transition (MZT; **b–e**). *grhl3* mRNA is rapidly downregulated, remaining primarily localised at the leading-edge margins (asterisk, **c**). (**g**) Sequence details of the exon 4 region of *grhl3*, showing a loss of 3 bp and insertion of a STOP codon cassette comprising 17 bases, resulting in a net gain (genomic lesion) of 14 bp in the *grhl3* mutant. (**h**) Schematic of the zebrafish *grhl3* protein (to scale; amino acids 1–557), showing trans-activation (TA), DNA-binding (DNA), and dimerisation (DIM) domains, indicating site of genomic lesion at codon 143. (**i**) Sequencing of WT and *grhl3* ^*−/−*(+*14bp*)^ embryos confirming presence of the genomic lesion. The red underline indicates (in WT) the position of the three deleted nucleotides, green underline shows insertion of 17 bp of STOP-cassette, black underline shows the resultant in-frame stop codon, yellow arrowhead indicates the CRISPR/Cas9-cut site. (**j**) RT-PCR showing ~50% loss of *grhl3* transcript in *grhl3* ^*−/−*(+*14bp*)^ embryos relative to controls at 8 hpf; a full-length gel photo is presented in Supplementary Figure S8. (**k**–**l**) Epiboly is delayed in *grhl3* ^*−/−*(+*14bp*)^ embryos (**l**) relative to WT (**k**), resulting in premature contraction of the leading-edge actin ring (arrows in L) at ~75% epiboly. (**m**,**n**) WT embryo at the onset of somitogenesis showing normal development (**m**), and *grhl3* ^*−/−*(+*14bp*)^ embryo (**n**) showing rupture of the EVL resulting in lethality (arrow in **n**). (**O**) Q-RT-PCR showing decreased *grhl3* expression, no change in *grhl2a*, and slightly elevated expression of *grhl2b* at ~75% epiboly in *grhl3* ^*−/−*(+*14bp*)^ embryos relative to WT controls.

Although MO-mediated loss-of-function transient knockdown zebrafish models have been reported previously for *grhl1* ^16,19^, *grhl2a* ^28^, *grhl2b* ^28,33^ and *grhl3* ^12^, thus far, only one loss-of-function genetic deletion model (a line null for *grhl2b*)^31^, has thus far been reported. In order to further investigate the role of this family, particularly *grhl3*, in zebrafish tissue patterning, organ development and morphogenesis, we firstly examined the early expression of *grhl3* by ISH (Fig. 1a–f). We found that *grhl3* is not maternally deposited, but rather is strongly and specifically activated in all EVL cells at ~4 hpf, corresponding temporally to the onset of zygotic transcription at the maternal-zygotic transition (MZT)^34^, before being downregulated in the EVL, but remaining expressed at high levels at the migratory front (“leading edge” of cells) during epiboly at 5 hpf. We generated a *grhl3* mutant fish via CRISPR/Cas9 mediated deletion and homologous integration, targeting Exon 4 (Fig. 1g) at a site which lay 3′ to seven putative in-frame ATG-initiation codons^35^, thereby minimising the chance of a 5′-truncated (yet still partially functional) protein being generated^35^. Our resultant mutant carries an insertion of a STOP codon cassette at codon 143 (Fig. 1h), that results in a net +14 bp gain within exon 4 of *grhl3* (confirmed by genotyping; Fig. 1i and Figure S7) and a stop codon being introduced at amino acid 143, with evidence of subsequent presumed nonsense-mediated decay (Fig. 1j). Double *grhl3* ^*−/−*(+*14bp*)^ mutant embryos displayed normal morphology until shield stage, and initiated epiboly at a timepoint equivalent to both WT and heterozygous siblings (Fig. 1k,l). However, we noted that epiboly then appeared slowed in *grhl3* ^*−/−*(+*14bp*)^ embryos compared to WT, with an initial bulging of the yolk cell that is retracted as epiboly continues, and culminating in a failure to complete epiboly and rupturing at the vegetal pole between ~80% epiboly and tailbud stage (Fig. 1m,n). This embryonic rupture is presumably due to significant weakening of the EVL/YSL interface. As expected, Q-RT-PCR analyses of ~80% epiboly stage WT and *grhl3* ^*−/−*(+*14bp*)^ embryos (prior to rupture) showed that transcript levels of *grhl3* were greatly reduced in phenotypic *grhl3* ^*−/−*(+*14bp*)^ embryos, with a concomitant decrease in the closely related family member *grhl1* (Fig. 1o). Transcript levels of the other two *grhl* fish orthologues, *grhl2a* and *grhl2b* were either unchanged (*grhl2a*) or slightly elevated (*grhl2b*) in *grhl3* ^*−/−*(+*14bp*)^ embryos, indicating firstly that *grhl3* may also regulate the expression of *grhl1* during epiboly and entry into gastrulation, recapitulating the rupture-phenotype reported previously following MO-mediated knockdown of both *grhl1* and *grhl3* ^36^, and secondly, that *grhl2b* may partially ameliorate early loss of *grhl3*.

### *grhl3* regulates midbrain-hindbrain neural tube folding

**Figure 2:**
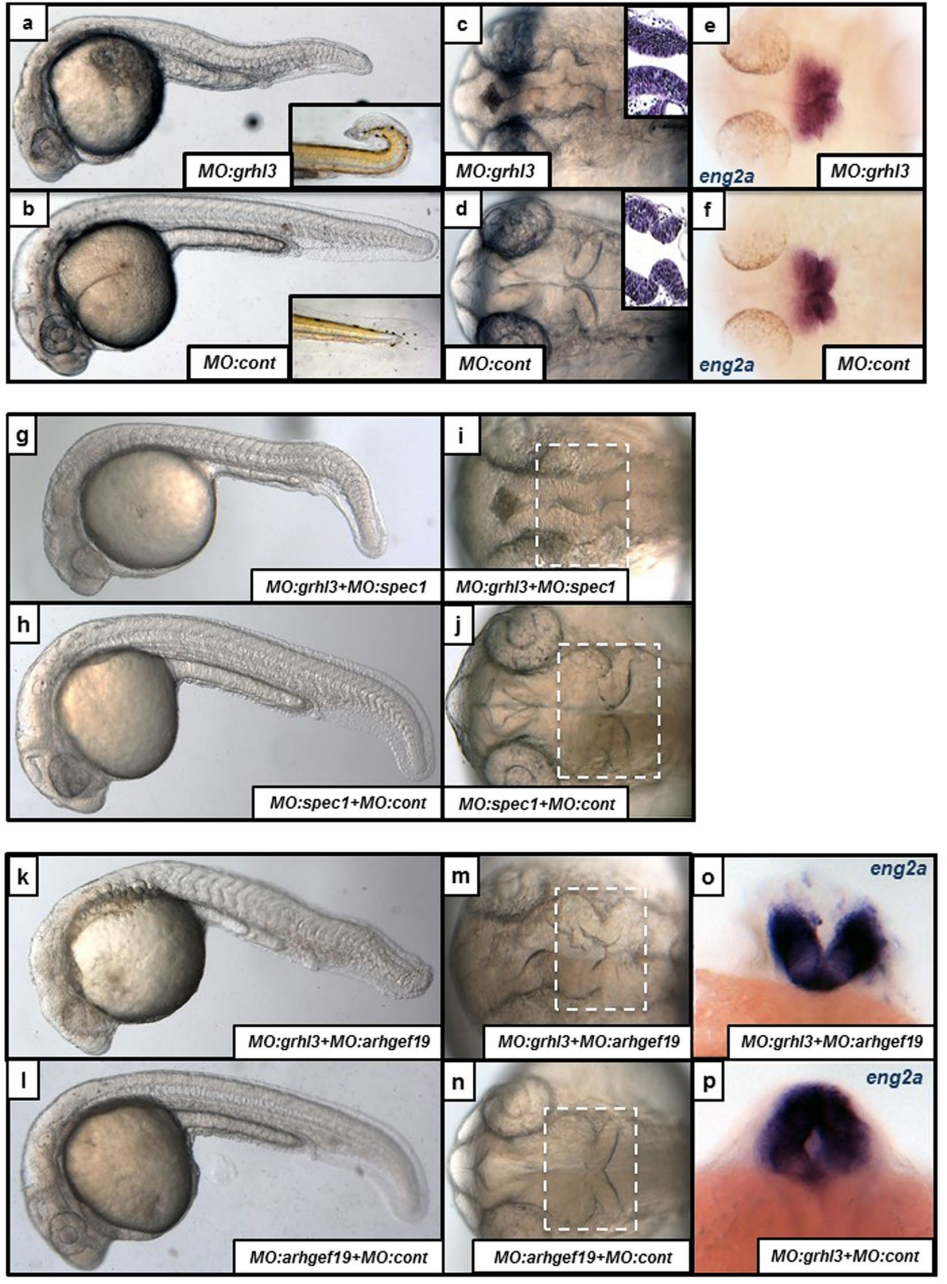
Midbrain-Hindbrain Boundary (MHB) morphogenesis is disrupted following knockdown of *grhl3*-dependent transcriptional networks. (**a**,**b**) Axial length is reduced, neural morphology is disrupted, and fish often exhibit a “curved-tail” phenotype (inset; 96 hpf) in MO:*grhl3* injected fish relative to controls. (**c**,**d**) The MHB defect in MO:*grhl3* injected fish presents as aberrant folding (dorsal view), confirmed by horizontal sections of H&E stained MHB tissue (**c,d**; inset). (**e,f**) Although the MHB is mis-folded, MHB patterning markers (such as *eng2a*) are not differentially regulated. (**g**–**j**) Following sub-phenotypic co-knockdown of *grhl3* and *spec1* (MO:*grhl3* + MO:*spec1*; **g,i**) the MHB is mis-folded when compared to phenotypically normal *MO:spec1* + *MO:control* injected fish (**h,j**). (**k**–**p**) Following sub-phenotypic co-knockdown of *grhl3* and *arhgef19* (MO:*grhl3* + MO:*arhgef19*; **k**), the fish are slightly shorter relative to phenotypically normal *MO:arhgef19* + *MO:control* injected fish (**l**). Furthermore, the neural tube exhibits an “open” phenotype at the MHB region (dorsal view; **m,n**); this is highlighted by ISH for the MHB marker *eng2a* (posterior view of MHB; **o**). relative to the phenotypically normal MHB seen in *MO:grhl3* + *MO:control* injected fish (**p**).

As genetic deletion of *grhl3* in zebrafish was embryonic lethal, we further investigated the role of *grhl3* using morpholino-mediated knockdown. As the *Grhl* family regulates both closure^9,10^ and morphogenesis^8,28^ of the neural tube, we injected 1–2 cell stage embryos with previously validated, specific antisense ATG- or splice blocking morpholinos (MO) directed against *grhl3* mRNA^12^, to examine putative changes in morphogenesis of the neural tube (Supplementary Table S1). Consistent with the morphological defects we had seen previously following MO-mediated knockdown of *grhl2b* ^28^, we noted an overall shortening of the axis (Fig. 2a,b), and the presence of a curved tail at 96 hpf (Fig. 2a,b; insets), analogous to the murine hypomorphic *Grhl3* mutant, *curly-tail* ^25^ which presents with posterior spinal cord defects. We also detected specific defects in the folding and morphogenesis of the midbrain-hindbrain boundary (MHB; Fig. 2c,d) by 24 hpf, without overt disruptions in the morphogenesis of other structures such as the eyes, heart, notochord or somites. These data were reminiscent of the MHB morphological changes we had observed previously following MO-mediated knockdown of *grhl2b* ^28^, indicating that the regulation of MHB morphogenesis by the *grhl* family may involve both multiple family members as well as conserved genetic mechanisms.

As MO-mediated knockdown of *grhl2b* in zebrafish also leads to loss of the key MHB patterning marker, and direct *grhl2b*-target gene *eng2a* (with subsequent loss of other MHB patterning markers and MHB formation)^28^, we speculated that MO-mediated knockdown of *grhl3* may similarly impact on MHB neural patterning. In order to determine whether this was the case, we investigated the expression of *eng2a* in *grhl3*-morphants. Somewhat surprisingly, we found no difference in the expression of *eng2a* (Fig. 2e,f) or expression of other MHB patterning markers, *pax2a*, *wnt1* or *her5* (Supplementary Figure S1) in *grhl3* morphants at any key stage of development examined (9 hpf, 18 hpf and 24 hpf), indicating that although *grhl3* function clearly regulates MHB morphogenesis, it does not impinge on neural patterning. These data are consistent with previous work showing that the processes of neural patterning and neural morphogenesis are mediated via distinct mechanisms^37–39^, and identify a novel specific regulator of MHB folding in the absence of aberrant neural patterning regulation.

### *grhl3* interacts with both *spec1* and *arhgef19* in MHB morphogenesis

Previous work from our groups had identified targets of the *Grhl*/*grhl* family involved in regulating both MHB morphogenesis in zebrafish (*spec1*)^28^ and cellular migration during epithelial wound-healing (*ARHGEF19*)^5^. To determine whether these downstream genes similarly co-operated with *grhl3* in regulating MHB morphogenesis in zebrafish, we performed knockdown experiments, whereby sub-phenotypic dosages of MO:*grhl3* were injected together with sub-phenotypic dosages of MOs targeting either *spec1* (Fig. 2g–j) or *arhgef19* (Fig. 2k–p). Consistent with previous experiments showing interaction between *grhl2b* and *spec1* in MHB morphogenesis^28^, we found that sub-phenotypic knockdown of *grhl3* and *spec1* together also led to slight axial shortening and aberrant MHB folding (Fig. 2g,i); sub-phenotypic knockdown of either MO:*grhl3* or MO:*spec1* together with MO:*control* resulted in no visible phenotypic perturbations (Fig. 2h,j; Supplementary Table S2). Although the constriction of the MHB in the *grhl3/spec1* double morphants appeared to initiate correctly, the bends did not fully form, possibly due to defective basal constriction^38^. Similarly, we found that sub-phenotypic knockdown of *grhl3* and *arhgef19* also led to mild axial shortening and aberrant MHB morphogenesis (Fig. 2k,m); sub-phenotypic knockdown of either MO:*grhl3* or MO:*arhgef19* together with MO:*control* resulted in no visible phenotypic perturbations (Fig. 2l,n; Supplementary Table S2).The *grhl3/arhgef19* double morphants also presented with an open dorsal neural tube, highlighted by ISH for the MHB marker *eng2a* (Fig. 2o,p). As *grhl3* is not expressed within the neural plate, rod or keel during embryogenesis^12^, but is expressed within the developing non-neural ectoderm/EVL (Supplementary Figure S2), this is a presumed secondary phenotype, most likely due to defects within the overlying formative epidermis. Furthermore, given the relatively small overlap between cells that would co-express both *grhl3* and *arhgef19* (within two discrete punctate regions of the EVL lateral to the MHB, and potentially in the EVL overlying the tip of the tail; Supplementary Figure S2), as well as *grhl3* and *spec1* (only within punctate regions of the EVL), significant differences in total transcript abundance by Q-RT-PCR analysis of both *arhgef19* or *spec1* in MO:*grhl3* injected fish were not seen (Supplementary Figure S2). Taken together, these data indicate that down-regulation of *grhl3* and *grhl3*-dependent signalling pathways leads to specific defects in neural morphogenesis, albeit not neural patterning.

### MO-mediated knockdown of *grhl3* signalling leads to differences in cell size and identity within the zebrafish EVL/periderm

**Figure 3:**
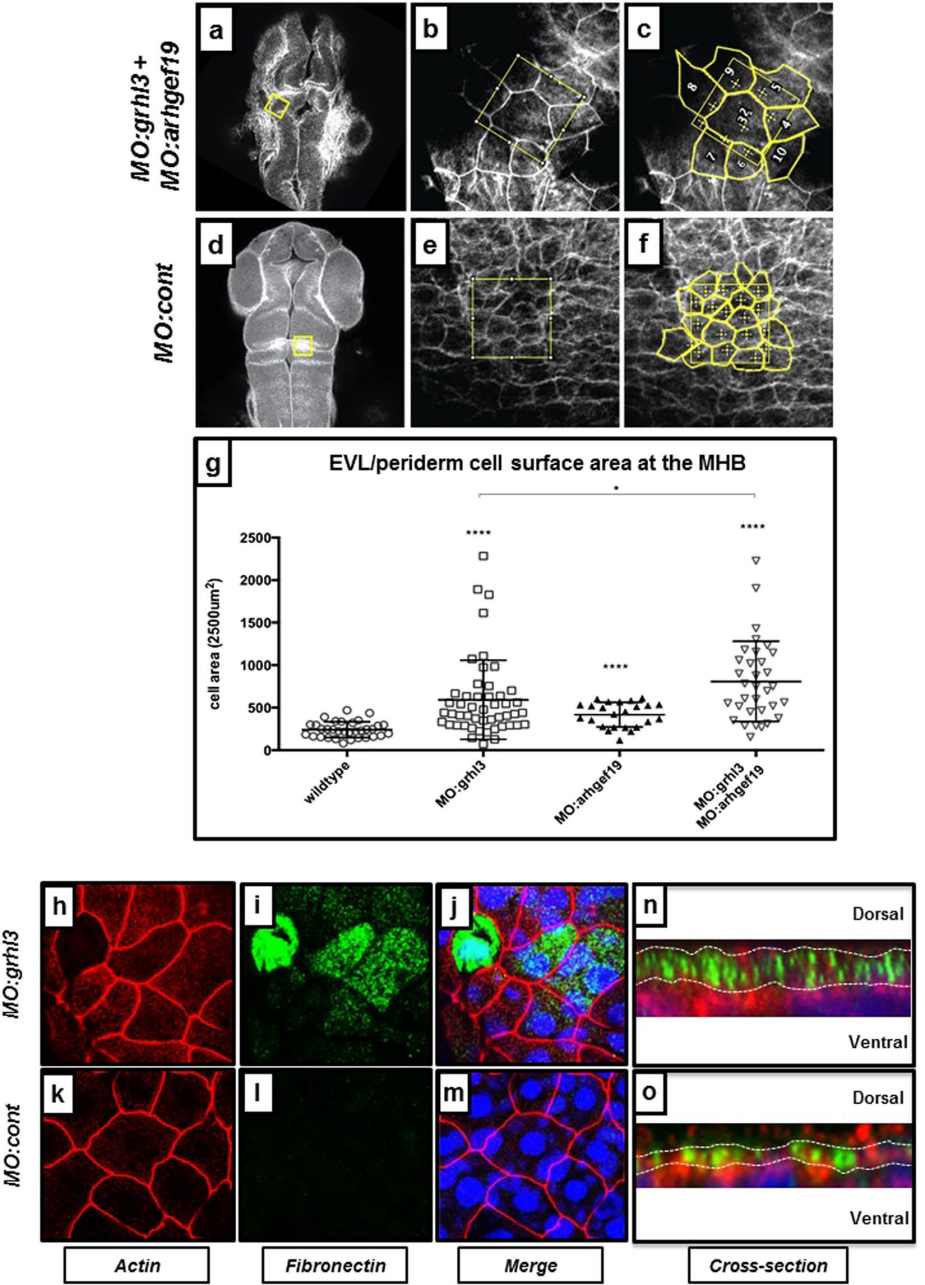
EVL cell size and identity is aberrant in *MO:grhl3* and *MO:arhgef19* injected zebrafish. (**a**–**g**) Rhodamine-phalloidin staining of F-actin in 24 hpf embryos injected with either *MO:grhl3* + *MO:arhgef19 MO:control* (**a,b**) or *MO:control* (**d**,**e**) shows that periderm cells with abrogated *grhl3/arhgef19* signalling are larger (boxed region in **b**,**e**). Morphometric quantitation of cell size using FiJi software (**c**,**f**) shows significant disparity in cell size in *MO:grhl3* + *MO: arhgef19* injected embryos (as well as singly-injected *MO:grhl3* and *MO: arhgef19* embryos), relative to controls (**G**). (**h**–**o**) The basal keratinocyte marker fibronectin (**i**) is ectopically expressed in EVL cells surrounding the MHB in MO:*grhl3*-injected embryos (merged image; **j**). Conversely, EVL cells (**k**) do not express fibronectin (**l**) in MO:*control* fish (merged image, **m**). Digital cross-section confirms that several dorso-ventral layers of fibronectin positive cells are present in MO:*grhl3*-injected fish (**n**), whereas fibronectin positive cells are distributed within a single, one-cell thick layer MO:*control* injected fish (**o**).

In order to analyse the *grhl3/arhgef19* phenotype in more detail, we performed confocal imaging of EVL cells at the level of the MHB in *grhl3* morphants, *grhl3/arhgef19* double morphants and control embryos at 24 hpf (Fig. 3a–f). In addition to confirming the MHB morphogenesis defects observed by low-power Brightfield microscopy, we extended these analyses to examine the EVL/periderm in greater detail. We characterised cellular morphology and cell size, cell fate, apoptosis, and marker gene expression (fibronectin, p63, ZO1 and E-Cadherin) to fully characterise EVL identity at 24 hpf in our *grhl3*-morphants. Morphometric quantitation of EVL/periderm cell size overlying the MHB showed that MO-mediated knockdown of either *grhl3* alone, or co-knockdown of both *grhl3* and *arhgef19* led to a highly significant increase in cell size (Fig. 3g); knockdown of *arhgef19* alone led to a smaller, yet still statistically significant increase in EVL/periderm cell size. These data indicate that *grhl3*-dependent pathways regulate EVL-developmental dynamics at the cellular level. In order to further investigate the characteristics of the EVL in *MO:grhl3*-injected fish, we performed immunohistochemical analysis of both actin (a marker of the cell-membrane of cells within the EVL) and fibronectin (a marker of the underlying basal keratinocytes) specifically within the outermost layer of the zebrafish EVL (Fig. 3h–m). As expected, actin was localised to the membrane of EVL cells in fish of both WT and *grhl3*-morphants. In WT fish, fibronectin is strongly expressed in basal keratinocyte cells at 24 hpf, but absent in the overlying EVL. MO-mediated knockdown of *grhl3* led to ectopic expression of fibronectin within EVL cells at 24 hpf, as well as an expanded pool of fibronectin-positive cells several layers thick (Fig. 3n,o), demonstrating that these cells express markers of multiple cell types. However, MO:*grhl3* EVL cells do not ectopically express the basal keratinocyte cell fate marker *p63* (Supplementary Figure S3), indicating that these EVL cells have not lost periderm identity, or acquired basal keratinocyte cell identity. *p63* is initially expressed in all epidermal progenitor cells at the 5 somite stage, but is later downregulated in EVL cells by 24 hpf^19^, indicating that *grhl3* is not required for the downregulation of *p63* in the EVL cells. Although we did detect a significant increase in apoptosis in *MO:grhl3* injected embryos, this was restricted to the region of the EVL and closely apposed tissue (Supplementary Figure S3), indicating this is a direct result of *grhl3*-knockdown which is likely to contribute to the neural tube phenotype. No significant differences were detected in expression of ZO-1 at either 8 hpf (Supplementary Figure S4) or 24 hpf (Supplementary Figure S5), or epithelial integrity (*cdh1* expression; Supplementary Figure S6). Taken together, our data show that MO-dependent inhibition of *grhl3*-signalling within the EVL leads to defects through production of fewer, larger cells, without concomitant disruption in cell fate, EVL integrity, cell death or establishment of junctional complexes. These data support the hypothesis that loss of *grhl3* in EVL cells results in the expression of components found in other epithelial cells without a change in cell fate, suggesting that *grhl3* functions to prevent EVL cells from expressing markers of other epithelial cell types.

### MO-mediated knockdown of both *grhl2b* and *grhl3* causes defects consistent with defective convergence-extension

**Figure 4:**
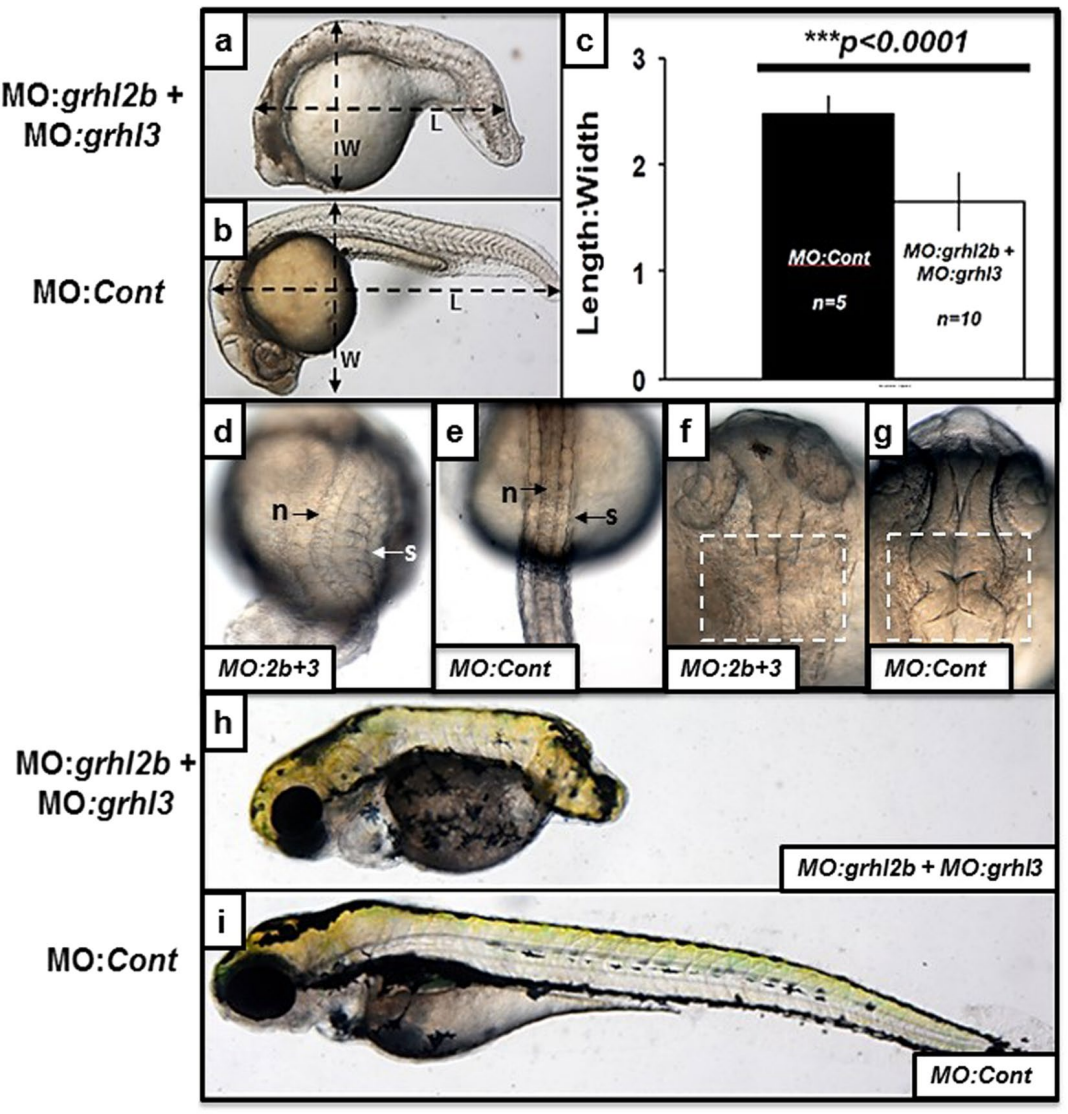
*grhl2b/grhl3* double morphants present with phenotypes highly consistent with disrupted convergence-extension mediated migration, as well as MHB folding defects. (**a**–**i**) *MO:grhl2b* + *MO:grhl3* injected double morphants (**a**) display a reduced axial length and decreased length:width ratio (LWR) relative to embryos injected with *MO:control* (**b**). These differences were quantitated (**c**), showing significantly decreased LWR in *MO:grhl2b* + *MO:grhl3* injected double morphants (p < 0.0001 by Student’s T-test). (**d**–**g**) Dorsal views of *MO:grhl2b* + *MO:grhl3* double morphants (**d**,**f**) relative to *MO:control* embryos (**e**,**g**) at 24 hpf highlighting the thicker trunk, broadened notochord (n) and splayed somites (s) characteristic of embryos with disrupted convergence-extension; as well as disruption of MHB morphogenesis (compare **f** with **g**). (**h**–**i**) *MO:grhl2b* + *MO:grhl3* double morphants (**f**) relative to *MO:control* embryos at 96 hpf, highlighting severely reduced axial extension.

To investigate the putative co-operative roles of *grhl2b* and *grhl3* in early patterning and gastrulation, we performed co-knockdown experiments, utilising our previously published morpholinos targeting both orthologues^12,28^. Although our previous studies of *grhl2b* or *grhl3* knockdown did not indicate that loss of either gene led to any appreciable defects in axial patterning, we found that co-injection of both MOs (each at phenotype-causing dosages) resulted in significant defects consistent with aberrant CE-mediated axial patterning (Fig. 4a,b; Supplementary Table S3). By quantitating these CE-like defects using the length to width ratio (LWR) measurement previously described for such analyses^40^, we found that these double morphants exhibited a ~40% decrease in LWR at 24 hpf (Fig. 4c), and furthermore, these fish presented with concomitant broadened somites and notochord (Fig. 4d,e), both classic hallmarks of the CE-mutant phenotype seen in models such as *trilobite*, *knypek* or *silberblick* ^40^; moreover, these double morphants also presented with significant defects in MHB morphogenesis (Fig. 4f,g). This phenotype was entirely consistent with the short, squat stature following *Grhl3* deletion, and occasional cranial exencephaly at the MHB region previously observed in the *Grhl3* ^*−/−*^ mouse mutant model^9^, and the CE-like phenotype common for animal models with impaired gastrulation movements during germinal layer formation. The CE-like defect was even more pronounced at 96 hpf (Fig. 4h,i), with fully-penetrant failure of posterior trunk extension in the most severely-affected double morphants. These data suggest that although MO-mediated knockdown of *grhl2b* or *grhl3* in isolation does not affect CE-movements, loss of both these factors in fish appear to lead to severe impairments in CE-dependent migration post gastrulation.

### Over-expression of *grhl3* causes defects in axis extension and also leads to axial duplication

**Figure 5:**
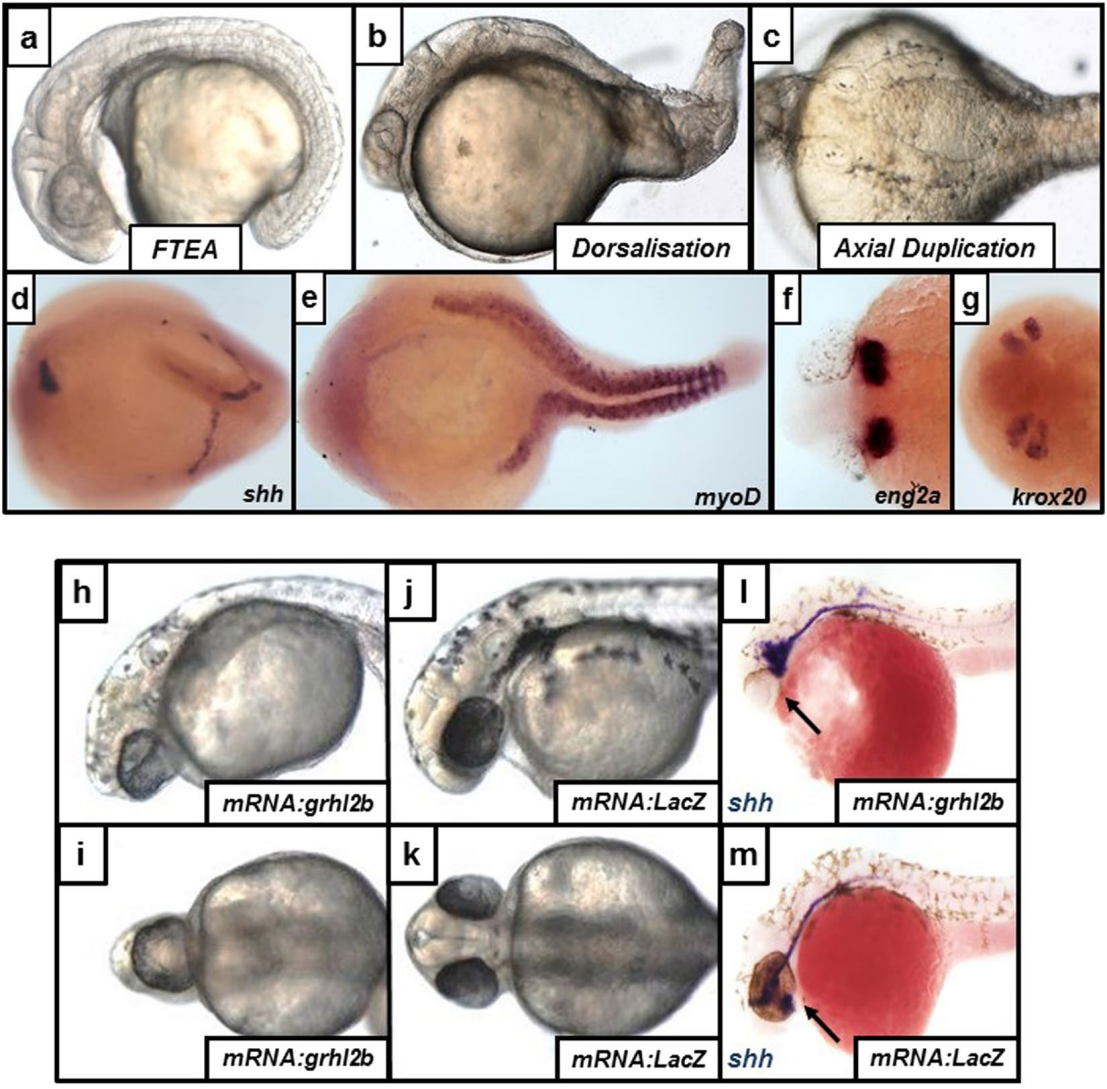
Over-expression of both *grhl3* and *grhl2b* results in defective patterning and cellular migration. (**a**–**g**) Over-expression of *grhl3* results in three disparate phenotypes, notably a failure to extend axis (FTEA; **a**), dorsalisation (**b**), or mid-axial duplication (**c**). ISH showing duplication or bifurcation of the neural tube (*shh*; **d**), somites (*myoD*; **E**), MHB (*eng2a*, **f**) and hindbrain rhombomeres (*krox20*; **g**) (**h**–**m**) Over-expression of *grhl2b* mRNA (lateral view H; ventral view, **i**) results in cyclopia, a defect not seen following injection of control (*LacZ*) mRNA (lateral view **J**; ventral view, **k**). This defect is likely due to impaired anterior neural tube extension in embryos over-expressing *grhl2b* mRNA, as shown by expression of the neural tube marker *shh* (arrow) by ISH (**l**–**m**).

We complemented our loss-of-function analyses with an analysis of the consequences on embryogenesis of over-expression of *grhl2b* or *grhl3*. To further support the strong evolutionary conservation of these factors, we also over-expressed full-length mRNAs of the murine orthologues, *Grhl2* and *Grhl3* respectively, with identical phenotypic consequences (Supplementary Table S4; Supplementary Table S5). Our micro-injection experiments over-expressing full length *grhl3/Grhl3* yielded three consistent, yet partially disparate phenotypes (dorsalisation, failure to extend axis and axial duplication; Fig. 5a–c). The first resulted in fish with normally developed anterior structures, including the MHB, but a severely shortened trunk and tail, which remained wrapped around the yolk rather than elongating, a phenotype we termed “failure to extend axis” (FTEA; Fig. 5a). The second phenotype was reminiscent of dorsalisation, with a severe reduction or absence of posterior structures (Fig. 5b). The third phenotype resulted in duplications of the trunk, reaching as far anteriorly as the MHB, and as far posteriorly as the mid-somite region (Fig. 5c), although interestingly, fish with either duplicated anterior (two heads) or posterior (two tails) regions were not seen. Molecular analyses on the axial duplication phenotype by ISH showed that both axes appeared to be patterned correctly (Fig. 5d–g), expressing markers of the posterior neural tube (*shh*), somites (*MyoD*), MHB (*eng2a*) and hindbrain (*krox20*), suggestive of either an axial duplication event or an exact symmetrical midline-bifurcation, comprising cells derived from all three germinal layers.

### Over-expression of *grhl2b* causes defects in eye-field separation and cyclopia

Fate mapping of cells involved in normal bilateral eye development indicate that the eye-field primordia undergoes significant rearrangement to accommodate the ventral diencephalon, putatively as a consequence of physical anterior migration of the neural tube, or through induction of localised diencephalic tissue to bisect the eye-field^41^. Over-expression of *grhl2b/Grhl2* yielded a striking phenotype of defective forebrain formation with concomitant cyclopia (Fig. 5h–k). This phenotype is reminiscent of other models of cyclopia, particularly in fish with defective PCP/CE signalling^40,42^ which is caused by the aforementioned defective anterior migration of the neural tube. To investigate whether cyclopia in our model was a result of failed neural tube migration, we performed ISH for the neural tube marker, *shh* (Fig. 5l,m). We found that *shh* expression in the *grhl2b*-overexpressing fish did not extend as far anteriorly as in *mRNA:LacZ* injected controls, and the diencephalic anlagen demonstrated a bunched and clustered appearance, highly consistent with impaired migration, rather than lack of neural tube formation, resulting in subsequent failure of eye-field separation. These data indicate that tight homeostatic regulation of *grhl2b* is essential for anterior migration of the zebrafish neural tube, and suggesting that *grhl2b* may negatively regulate, or suppress, a factor critical for neural tube migratory dynamics.

## Discussion

We present here several novel phenotypes associated with disrupted *grhl*-family signalling in the zebrafish, *Danio rerio*, following both MO-mediated down-regulation, and over-expression through injection of CpG-capped full-length mRNA. Both loss or gain of function models showed specific effects on cellular migration, cellular dynamics, morphogenesis, and axial patterning/extension, thereby greatly expanding upon the known roles of the *Grhl* family during vertebrate embryonic development.

When *grhl3* is down-regulated, either alone or together with downstream targets *spec1* or *arhgef19*, the folding (but critically not the neural induction or subsequent patterning) of the midbrain-hindbrain boundary (MHB) is disrupted. These data are consistent both with previous work from our laboratory showing disrupted neural tube closure in mice lacking *Grhl2* or *Grhl3* ^9,10^, and aberrant MHB folding following loss of *grhl2b* ^28^, as well as the newly-emerging paradigm which separates the processes of neural tube morphogenesis from induction and patterning^37–39^. Although the *grhl3/arhgef19* phenotype is consistent with the open posterior neural tube and occasional exencephaly of *Grhl3* loss in mouse^9^, the mechanisms of neurulation in fish (evagination of a lumen from a solid rod of cells) differ from those in mouse, suggesting that this common phenotype is either “fated” during the convergence-extension movements of the pre-tubular neural plate, which is common to both species^43^, or more likely, is caused through distinct mechanisms.

In the context of neural tube closure, *Grhl3* in mouse is expressed within the non-neural ectoderm (NNE) immediately abutting the neural plate border preceding neurulation^9^, and this NNE is critical for neurulation^44^. It is likely that loss of *Grhl3* in this region in mouse leads to either mechanical, signalling, extra-cellular matrix (ECM) deposition or polarity defects in the NNE which impinge on the subsequent co-ordinated migration, apposition and closure of the neural tube. The role of the ECM between the neural plate and its surrounding tissue layers during neural tube morphogenesis is well-established. Knockdown of the ECM components, laminin and fibronectin disrupt neural tube morphogenesis in zebrafish by preventing tissue apposition and coupling movements between the neural plate and mesodermal tissues^45^. The expression of *grhl3* in the developing zebrafish is more abundant than in mouse, present throughout the entire EVL/periderm overlying the neural plate (but importantly, not within the neural plate itself) up to and including neural tube closure, ventricle inflation and morphogenesis^12,46^. Given that our results identified an altered expression pattern of the ECM component fibronectin in *grhl3*-morphants, it is possible that changes in ECM composition (together with the concomitant increase in apoptosis we observed) in *MO:grhl3* embryos cause a disruption and subsequent remodelling of the tissue coupling between the EVL and neural plate, resulting in a loss of anchoring, and may be responsible for the MHB defects observed. Consistent with our data showing aberrant EVL/periderm cell size and morphology in *grhl3* and *grhl3/arhgef19*-morphants, and early rupture of the *grhl3* ^*−/−*(+*14bp*)^ embryos, it is likely that the EVL in these fish is significantly weakened. As the *grh/Grhl/grhl* family are crucial regulators of both cell division and survival^6,12,28,47^, the increase in cell size is likely due to decreased proliferation or increased apoptosis of the EVL in early development, resulting in fewer cells forming the overlying EVL/periderm barrier during neurulation. This suggests that this tissue may possess insufficient tensile strength to efficiently contain the expanding neural tube during evagination – hence, a “rupture” of the neural tube at its dorsal-most point, resulting in an open-neural tube. This phenotype is reminiscent of the zebrafish mutant *bloody-fingers; blf* ^48^, whereby a defective overlying epidermal layer results in delamination of dorsal neural tube cells and a spina bifida like phenotype. We have also recently noted a similar impact of the NNE and/or ECM-remodelling defects in *Grhl3* ^*−/−*^ mice at the level of the cranial neural tube, and in particular the influence of the overlying epidermis on the development of the skull; namely, these mice present with an increased apposition of the frontal and parietal bones during development in a phenotype analogous to the human condition craniosynostosis^49^.

Although our novel *grhl3* ^*−/−*(+*14bp*)^ fish line does not allow analysis of tissue patterning and development, due to the very early embryonic lethality, it is likely to be an excellent model of the maternal-zygotic transition (MZT). Previous work in *Drosophila* indicates that *grh* regulates activation of the zygotic genome^50^, whereas unbiased large-scale molecular profiling of MZT-stage zebrafish embryos indicates that *grhl3* is the fourth most-highly upregulated transcript of the earliest zygotically-transcribed genes^51^. Interestingly, *blf* is the sixth-most highly upregulated transcript, suggesting there may be commonalities between genetic regulation during the MZT, EVL development, and epidermal/neurulation defects.

Both our MO data and our striking mutant phenotype raise the question as to whether other *grhl*-orthologues may be functionally compensating for *grhl3* in early development. Animal models in which *Grhl1* is deleted or downregulated do not present with a neurulation phenotype^17–19^, moreover we show here that *grhl1* is not upregulated to compensate for *grhl3* loss (Fig. 1o). Although little is known about *grhl2a*, we show here that *grhl2a* expression is also largely unchanged (Fig. 1o). We have previously shown that the earliest expression of *grhl2b* is in the leading edge of the neural plate, and/or the polster, from approximately 8 hpf^28^; moreover both our previous work, plus another study^33^, do not show any role for *grhl2b* in epidermal/EVL development. Our data therefore allow us to conclude that loss of *grhl3* in our mutant is not significantly functionally compensated for by up-regulation of other *grhl*-family members. Together, these finding suggest that *grhl3* and its downstream target genes regulate neural tube morphogenesis as a secondary consequence of defective EVL/periderm/epidermis development and maintenance.

*Grhl3* interacts genetically with mammalian Planar Cell Polarity (PCP) genes^5^, and correct planar cell-polarity establishment is required for subsequent cellular migration, especially during convergence-extension (CE) mediated cell movements during axial patterning and morphogenesis. Given this, an interesting observation is the fact that although *grhl3*-morphants are often shorter than controls, they do not display an obvious CE-defect, unlike morphants in which both *grhl2b* and *grhl3* have been concomitantly down-regulated. Although this may appear to be at odds with mouse *Grhl3*-knockouts, which are noticeably shorter and broader than WT-littermates^9^, as MO-mediated knockdown in fish does not lead to full inhibition of gene function, one may surmise that residual *grhl3* gene-function in zebrafish embryos is sufficient to preserve the normal polarity establishment and signalling required for correct CE migration. Unfortunately, CRISPR-mediated deletion of *grhl3* in the *grhl3* ^*−/−*(+*14bp*)^ embryos does not allow further examination of gastrulation phenotypes due to premature lethality during epiboly, which perhaps suggests a lower degree of redundancy between *grhl*-orthologues in zebrafish than in mouse. The *grhl2b/grhl3* double morphants however display the classic zebrafish CE phenotype of a shortened trunk, decreased length-to-width ratio, broadened notochord and thickened somites, and further strongly suggest that one role of *grhl2b* and *grhl3* interaction is to regulate convergence-extension in axial development. Furthermore, genetic co-operativity between these genes has previously been described in mammalian models - insertion of *Grhl2* into the *Grhl3* genomic locus led to rescue of a proportion of neural tube defects^3^. The recent finding that mutations in human *Grhl3* are associated with spina bifida^52^, in itself a defect of neurulation possibly due to incorrect convergence-extension, suggests that zebrafish *grhl2b/grhl3* double-morphants may ultimately prove to be a useful model of human CE-like defects.

Our over-expression data show further novel gastrulation phenotypes associated with disrupted *Grhl*-gene signalling. Increased *grhl2b* expression leads to failed anterior neural tube migration and cyclopia. These defects are reminiscent both of over-expression of the homeobox transcription factor, and direct *grhl2* target gene *engrailed 2a*, which suppresses formation of anterior structures, including eyes and forebrain^53^, and over-expression of *slit2*, the ligand for *roundabout* (*robo*) which also results in failed neural tube migration and cyclopia^54^. Our Q-RT-PCR analyses, however showed that neither gene was significantly differentially regulated in *grhl2b* over-expressing morphants (data not shown). Other pathways which may be involved include *nodal*, *shh*, *zic2* ^55^ and the nodal-related protein *squint* ^56^, and future work will focus on analysing these, and other mechanisms of failed anterior neural tube migration.

Three further novel phenotypes were seen following over-expression of *grhl3*, although interestingly, no specific defects in MHB morphogenesis or neurulation were detected. These data support the hypothesis of a weakened EVL overlying the periderm; whereas loss of *grhl3* leads to loss of EVL integrity, gain of *grhl3* does not lead to any further increase in EVL tensile strength or integrity. All of dorsalisation, failure to extend axis (FTEA) and midline bifurcation are highly consistent with defective cellular migration. The FTEA phenotype in particular suggests a milder dorsalisation phenotype, as although the anterior structures are patterned correctly, ventral structures are under-represented, in this case due to an extension defect. The mechanism for these phenotypes is currently unknown, although an attractive possibility is the involvement of a newly-characterised *Grhl*-family target gene, *E-Cadherin* ^27^. Severely affected zebrafish mutants lacking *e-cadherin* exhibit a highly-similar FTEA phenotype^57^ to our *grhl3* over-expressing fish, and it is a possibility that defective ECM scaffolding through repressed *e-cadherin* may contribute to failed cellular migration and subsequent tail extension. Furthermore, recent studies indicate that in addition to activating *E-Cadherin*, or serving to alter *E-Cadherin* localisation^58^, *Grhl3* may also act as an *E-cadherin* suppressor^59^, indicative of a dichotomous relationship between these two factors, which together may regulate convergence-extension mediated movements during embryogenesis.

The third phenotype, that of midline bifurcation in our model is of the rarer variety that doesn’t involve either head duplication, (e.g. in Xenopus over-expressing *wnt*)^60^ or tail duplication (e.g. embryos over-expressing *activin*)^60^, but rather a specific duplication which occurs in the trunk. Interestingly, similar axial duplication is also seen in embryos overexpressing *fgf8* ^61^, itself a known upstream regulator of the *grh/grhl* family^28^. These data add further support to the theory that *Fgf*-signalling may be regulate the phosphorylation and resultant function of the *grh/Grhl* genes across broad developmental contexts, having been previously experimentally determined for *grh* in Drosophila tracheal branching^62^ and regulation of MHB morphogenesis and neuroblast survival in conjunction with *grhl2b* in zebrafish^28^.

Taken together, our study characterises numerous novel vertebrate phenotypes due to disrupted *grhl*-signalling, and highlights the remarkable diversity of phenotypic processes regulated by this gene family. Future work will focus on further elucidating the genetic pathways (both upstream and downstream of the *grhl*-family) by which the vertebrate *grhl* family regulates these myriad developmental regulatory effects.

### Data Availability Statement

All data (including raw data and supplementary data), as well as the *grhl3* ^*−/−*(+*14bp*)^ fish line will be made available upon request.

## Electronic supplementary material

Supplementary Material
