# Supplementary material for: Mis-expression of grainyhead-like transcription factors in zebrafish leads to defects in enveloping layer (EVL) integrity, cellular morphogenesis and axial extension
Source: Sci Rep. 2017 Dec 14;7:17607. doi: 10.1038/s41598-017-17898-7 (PMC5730563; doi:10.1038/s41598-017-17898-7)
Supplement: Supplementary file 1 — Supplementary Material [file 41598_2017_17898_MOESM1_ESM.pdf]

Mis-expression of *grainyhead-like* transcription factors in zebrafish leads to defects in enveloping layer (EVL) integrity, cellular morphogenesis and axial extension.

Lee B. Miles[1], Charbel Darido[2], Jan Kaslin[3], Joan K. Heath[4], Stephen M. Jane[5,6], Sebastian Dworkin[1#],

[1]Department of Physiology, Anatomy and Microbiology, La Trobe University, Bundoora, VIC, 3086

[2]The Victorian Comprehensive Cancer Centre, Peter MacCallum Cancer Centre, Parkville, VIC, 3050.

[3]The Australian Regenerative Medicine Institute, Monash University, Clayton, VIC, 3168.

[4]Department of Chemical Biology, The Walter and Eliza Hall Institute, Parkville, VIC, 3050, Australia

[5]Department of Medicine, Monash University Central Clinical School, Prahran VIC 3181, Australia.

[6]Department of Hematology, Alfred Hospital, Prahran VIC 3181, Australia.

#Author for correspondence: s.dworkin@latrobe.edu.au

Fig. S1: The expression of Midbrain-Hindbrain Boundary (MHB) patterning marker genes is unchanged in MO:*grhl3* injected fish.

The expression profile of MHB patterning marker genes *eng2a, her5, pax2a* and *wnt1* is largely unchanged in MO:*grhl3* injected embryos relative to MO:*control* injected embryos at all stages of MHB development examined – 9-10 hpf; 18 hpf and 24 hpf.

Fig. S2: Expression patterns of *grhl3* and *arhgef19* in early zebrafish development.

(A-F) *grhl3* is expressed within the pharyngeal endoderm (PE) from 10 hpf (A), a timepoint at which expression within the EVL is also visible. Expression within both these regions persists until at least 18 hpf (B-C). *grhl3* expression is not seen in brain; non-specific probe trapping (C) is confirmed as artefact following both brightfield imaging of coronal sections (D) and subsequent H&E staining of both coronal (E) and sagittal (F) sections. Note persistent expression of *grhl3* within the PE (D-F) and occasional puncta of staining within the EVL (F). (**G-H**) Expression of *arhgef19* is seen in the EVL, lateral to the MHB (arrows, G), as well as in the EVL at the posterior-most region of the extending tail (arrow, H) at 24 hpf. (**I**) Q-RT-PCR showing expected non-significant decrease in *arhgef19* and *spec1* transcripts in *MO:grhl3* injected embryos.

Fig. S3: Characterisation of cell identity and viability in the EVL and basal layers of *MO:grhl3* injected fish. (A-H) DAPI staining showing no loss of nuclei in either EVL or basal layers (A-B). Rhodamine-Phalloidin staining showing normal distribution of F-actin in the EVL (C) and basal layer (D). There are no ectopic basal keratinocytes (*p63+*) present in the EVL (E), although, as expected, these are abundant in the basal layer (F; merged views G-H). (**I-K**) Activated Caspase-3 immunohistochemistry reveals few apoptotic cells in control fish (I) relative to the significantly increased numbers of apoptotic cells seen in *MO:grhl3* injected embryos (J) at the level of the midbrain and hindbrain (boxed region in I-J)**;** orthogonal views at the levels indicated (arrowheads; I-J) confirm that the majority of apoptotic cells are seen in the EVL and adjoining dorsal-most neural tissue, but are largely absent from the deeper neural tissue. These data are quantitated from n=4 controls and n=3 *MO:grhl3*-injected embryos (K).

Fig. S4: Characterisation of tight junction formation following knockdown of *grhl3* and *arhgef19* at 8 hpf. (**A-C**) The expression of ZO1 **(A)** and actin **(B)** in the EVL (**C**; merged) of WT fish is unchanged following MO-mediated knockdown of *grhl3* (D-F), *arhgef19* (G-I) or both *grhl3* and *arhgef19* together **(J-L)** at 8 hpf.

Fig. S5: Characterisation of tight junction formation following knockdown of *grhl3* and *arhgef19* at 24 hpf. (**A-C**) The expression of ZO1 **(A)** and actin **(B)** in the EVL (**C**; merged) of WT fish overlying the region of the midbrain-hindbrain boundary (MHB) is unchanged following MO-mediated knockdown of *grhl3* **(D-F)**, *arhgef19* **(G-I)** or both *grhl3* and *arhgef19* together **(J-L)** at 24hpf. Note increased EVL cell size in both *MO:grhl3* **(D-F)** and *MO:grhl3+MO:arhgef19* **(J-L)** injected embryos.

Fig. S6: Characterisation of epithelial integrity marker and *grhl*-target *gene E-cadherin* following modulation of *grhl3* and *arhgef19* activity at 24 hpf. (**A-C**) The expression of *E-cadherin* **(A)** and actin **(B)** in the EVL (**C**; merged) of WT fish overlying the region of the midbrain-hindbrain boundary (MHB) is unchanged following MO-mediated knockdown of *grhl3* **(D-F)**, *arhgef19* **(G-I),** both *grhl3* and *arhgef19* together **(J-L)**, or over-expression of *grhl3* mRNA **(M-O)** at 24 hpf. Note increased EVL cell size in both *MO:grhl3* **(D-F)** and *MO:grhl3+MO:arhgef19* **(J-L)** injected embryos. Nuclei in merged panels **(C, F, I, L, O)** are shown by DAPI staining.

Fig. S7: Genotyping of 5 separate *grhl3*$^{-/-(+14bp)}$ embryos. Extended sequence length from a representative wild-type embryo (WT) as well as 5 individual *grhl3*$^{-/-(+14bp)}$embryos at 80% epiboly (#1-#5), showing loss of GTC and insertion of TTAATTAAGCTGTTGTA (+3/-17; net insertion of 14bp; boxed region) in each of the *grhl3*$^{-/-(+14bp)}$embryos.

Fig. S8: Full size DNA electrophoresis gel of image presented in Figure 1J. Lane 1 shows 5μl of 1kb Quick Load Extend DNA Ladder (NEB; Cat# N3239S) and Lane 6 shows 5μl of 100bp Quick Load DNA Ladder (NEB; Cat# N0467L). Lanes 2-3 show *grhl3* transcript from control and *grhl3*$^{-/-(+14bp)}$ mutant embryos respectively at 80% epiboly; lanes 4-5 show *grhl3* housekeeping gene *ef1*α transcript from control and *grhl3*$^{-/-(+14bp)}$ mutant embryos respectively. This figure is a full-length gel of the cropped image in Fig. 1J.

Table S1: Phenotypic incidence and penetrance following *grhl3* knockdown using ATG and splice-blocking morpholinos. Quantitation of dose-dependent penetrance of MHB phenotypes following injection of varying doses of ATG and splice-blocking anti-*grhl3* morpholinos.

Table S2: Phenotypic incidence and penetrance following combinatorial knockdown of *grhl3, spec1* and *arhgef19.* Quantitation of dose-dependent penetrance of MHB phenotypes following knockdown of *grhl3, spec1* and *arhgef19*, together with appropriate controls.

Table S3: Phenotypic incidence and penetrance following combinatorial knockdown of *grhl2b* and *grhl3.*

MHB and CE phenotypes following knockdown of *grhl2b* and following injection of varying doses of morpholinos, together with appropriate controls.

Table S4: Quantitation of phenotypes observed following over-expression of *Grhl3* and *grhl3.* Quantitation of dose-dependent penetrance of phenotypes following injection of varying doses of both zebrafish (*grhl3*) and murine (*Grhl3*) mRNAs.

Table S5: Quantitation of phenotypes observed following over-expression of *Grhl2* and *grhl2b.* Quantitation of dose-dependent penetrance of phenotypes following injection of varying doses of wild-type (*grhl2*) and FLAG-tagged (*grhl2*-FLAG) zebrafish and murine (*Grhl2*) mRNAs.

Fig. S1

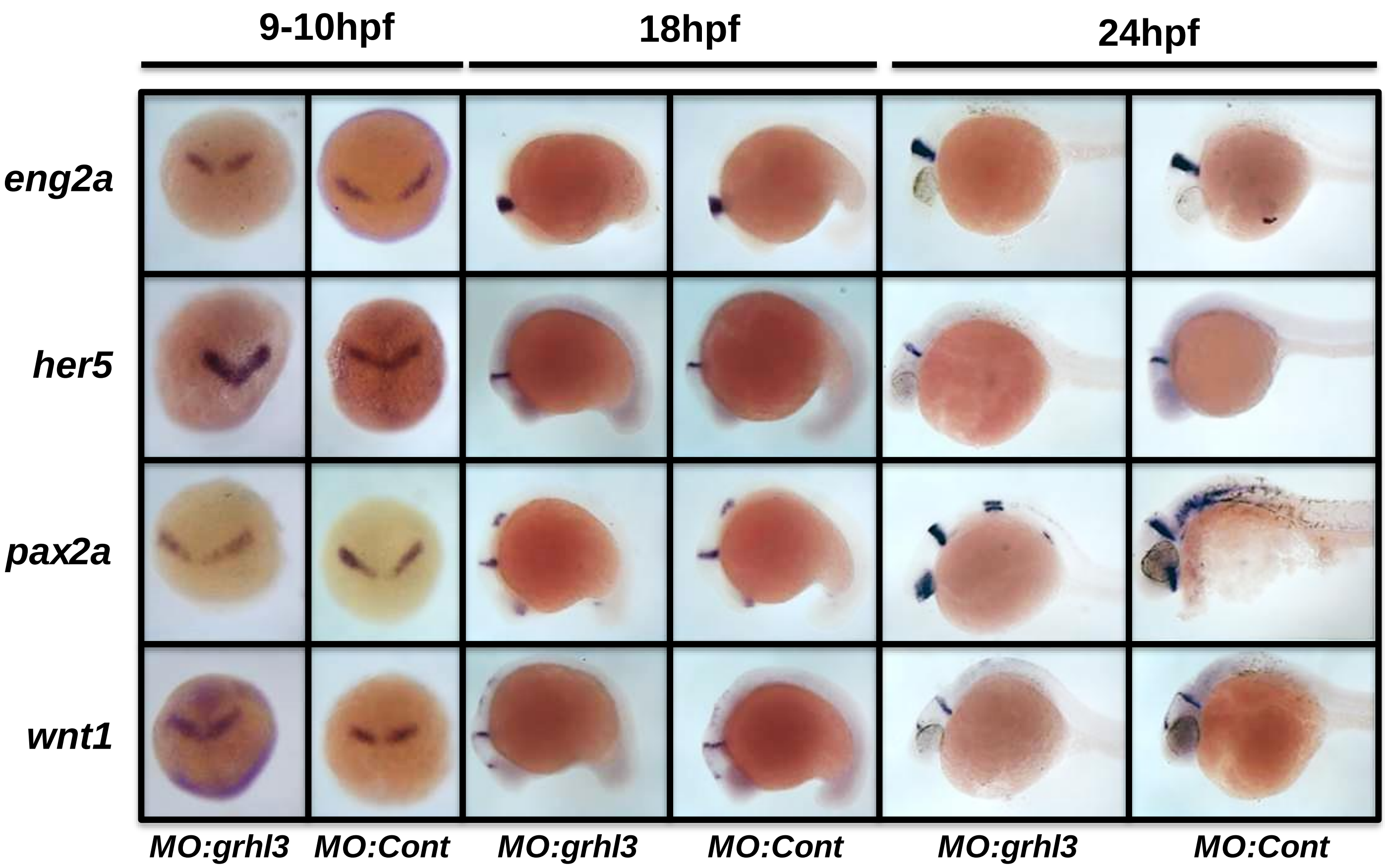

9-10hpf
18hpf
24hpf
eng2a
her5
pax2a
wnt1
MO:grhl3
MO:Cont
MO:grhl3
MO:Cont
MO:grhl3
MO:Cont

Fig. S2

grhl3 expression

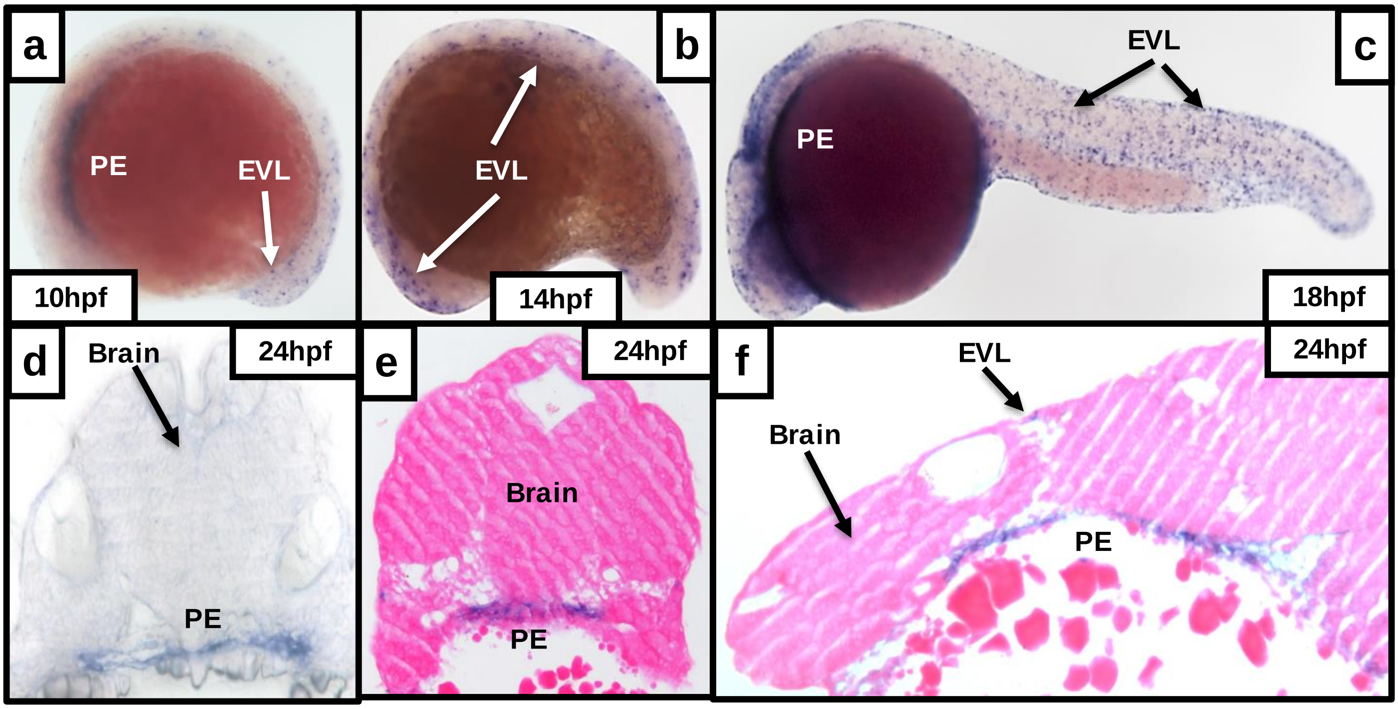

arhgef19 expression

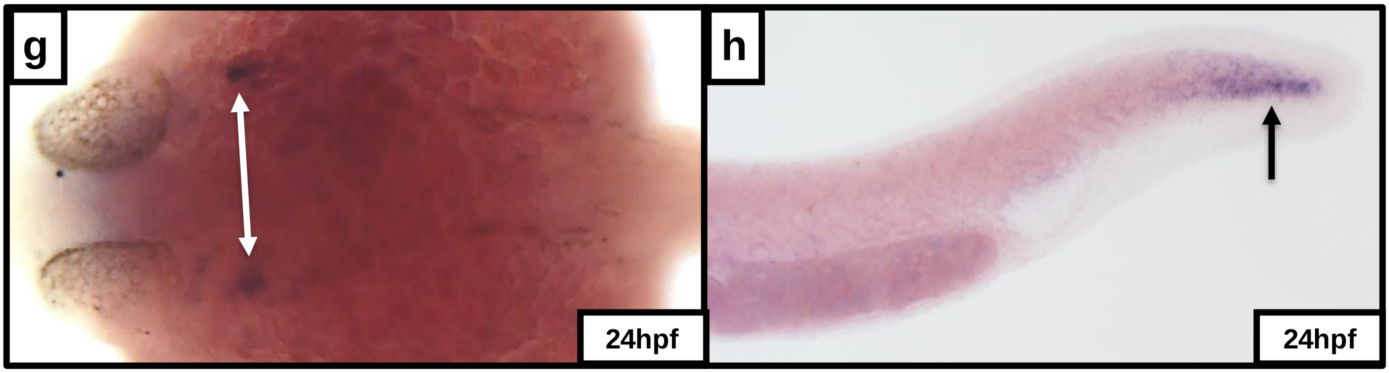

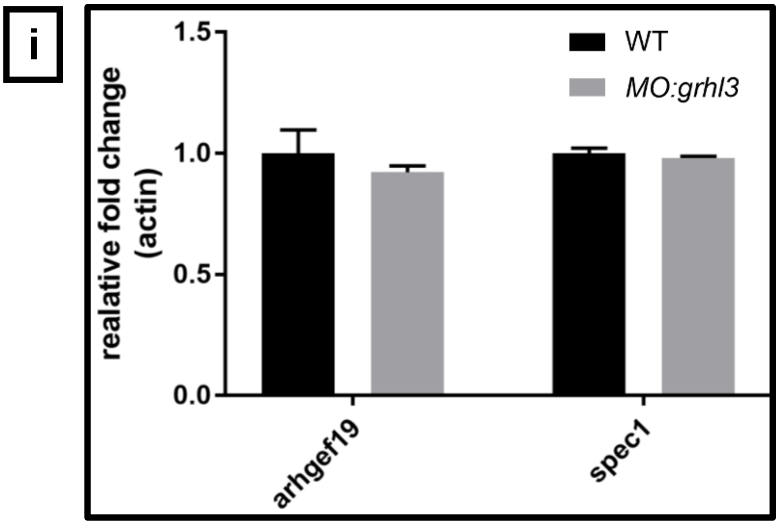

Fig. S3

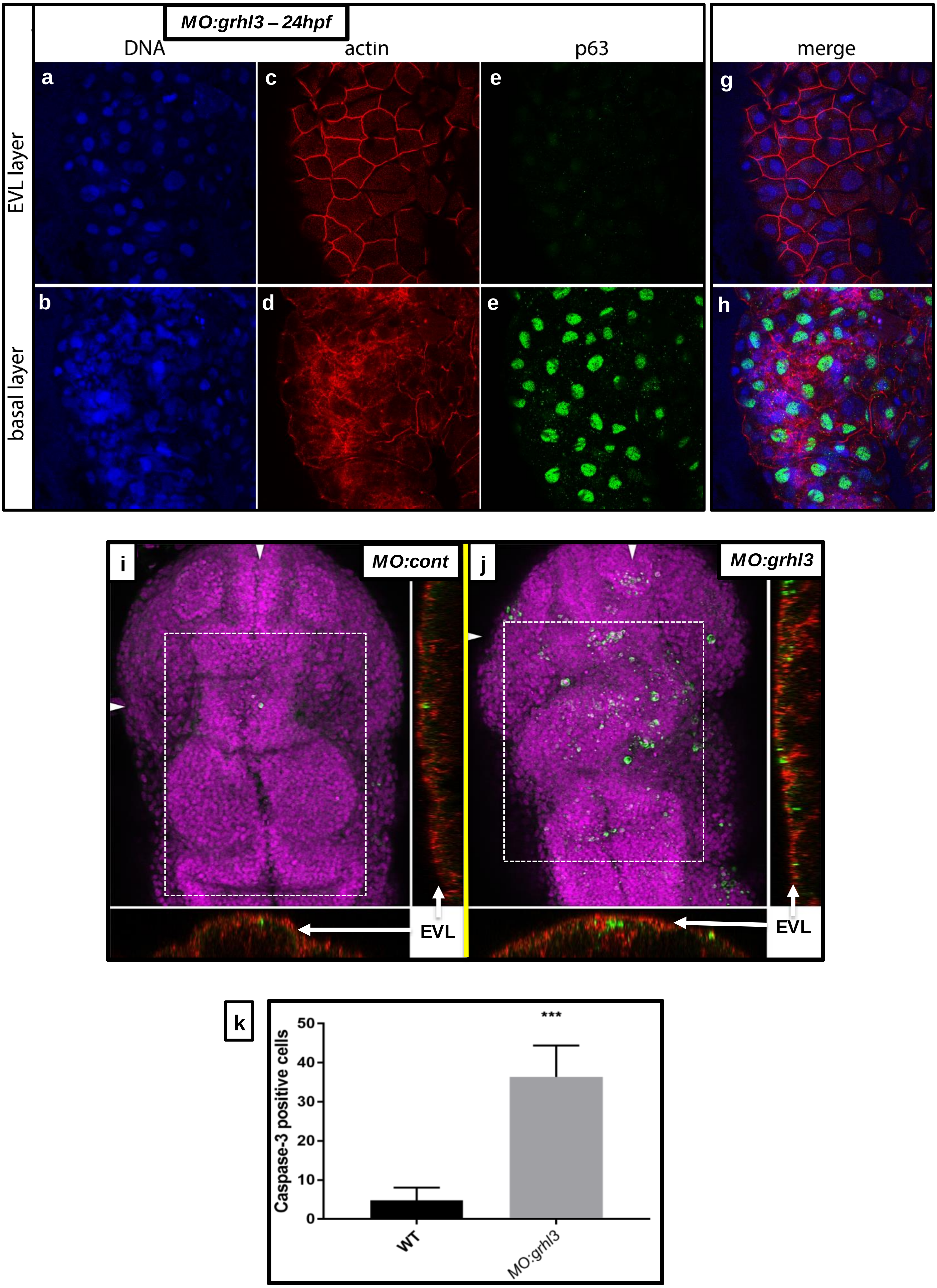

MO:grhl3 – 24hpf
DNA
actin
p63
merge
EVL layer
basal layer
a
b
c
d
e
e
g
h
i
MO:cont
j
MO:grhl3
EVL
EVL
k
Caspase-3 positive cells
50
40
30
20
10
0
***
WT
MO:grhl3

Fig. S4

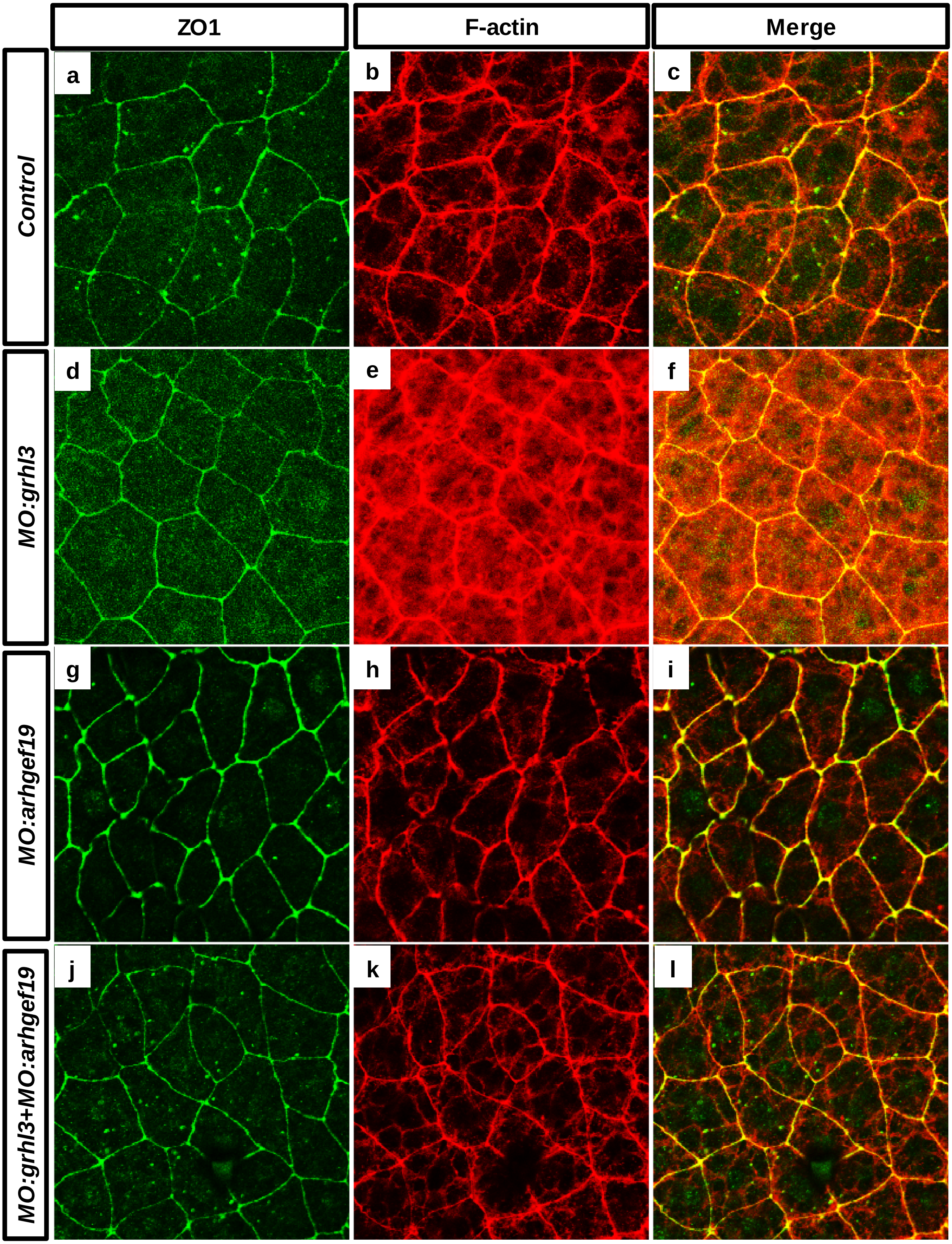
ZO1
F-actin
Merge
Control
MO:grhl3
MO:arhgef19
MO:grhl3+MO:arhgef19
a
b
c
d
e
f
g
h
i
j
k
l

Fig. S5

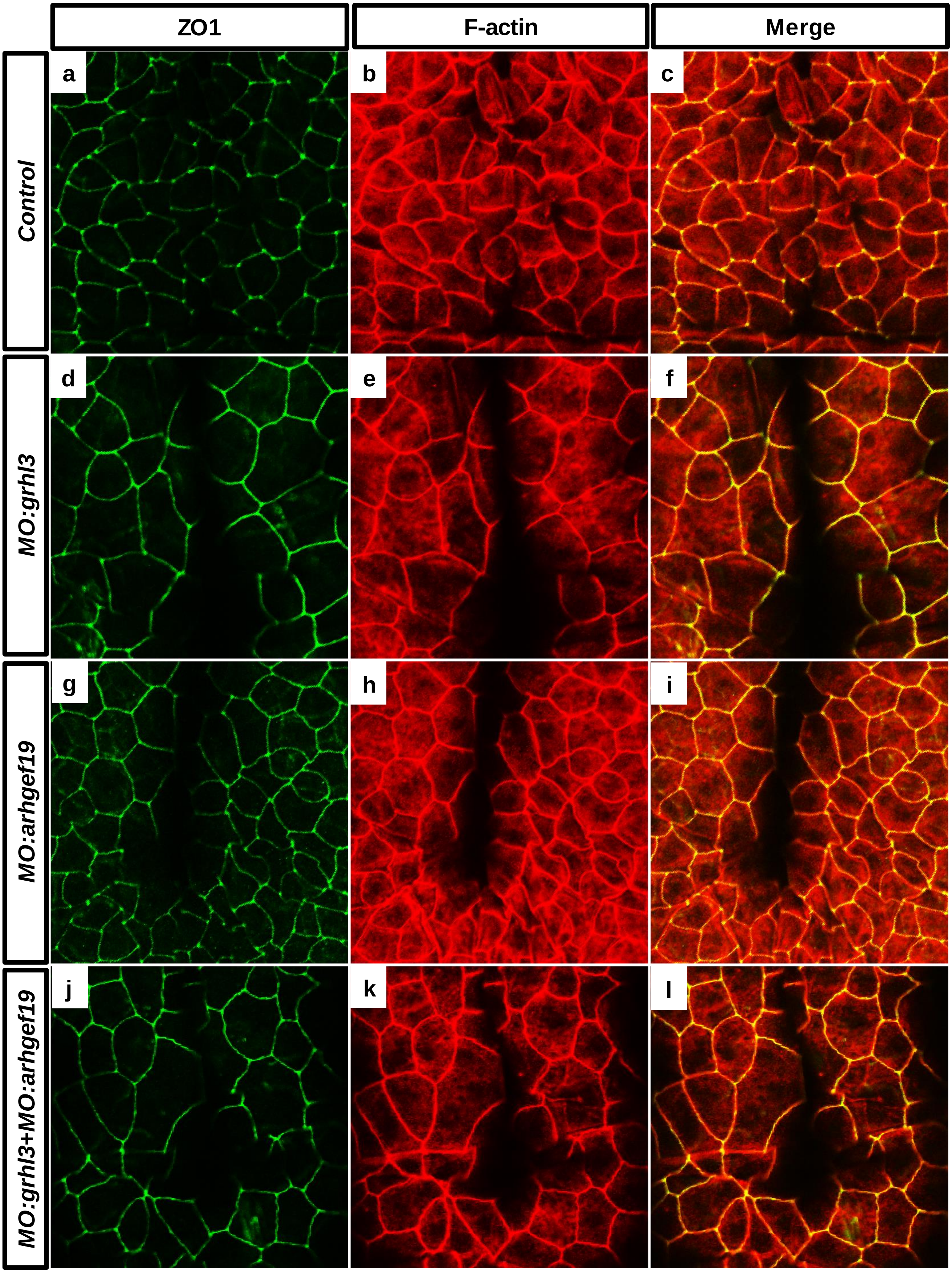
ZO1
F-actin
Merge
Control
MO:grhl3
MO:arhgef19
MO:grhl3+MO:arhgef19
a
b
c
d
e
f
g
h
i
j
k
l

Fig. S6

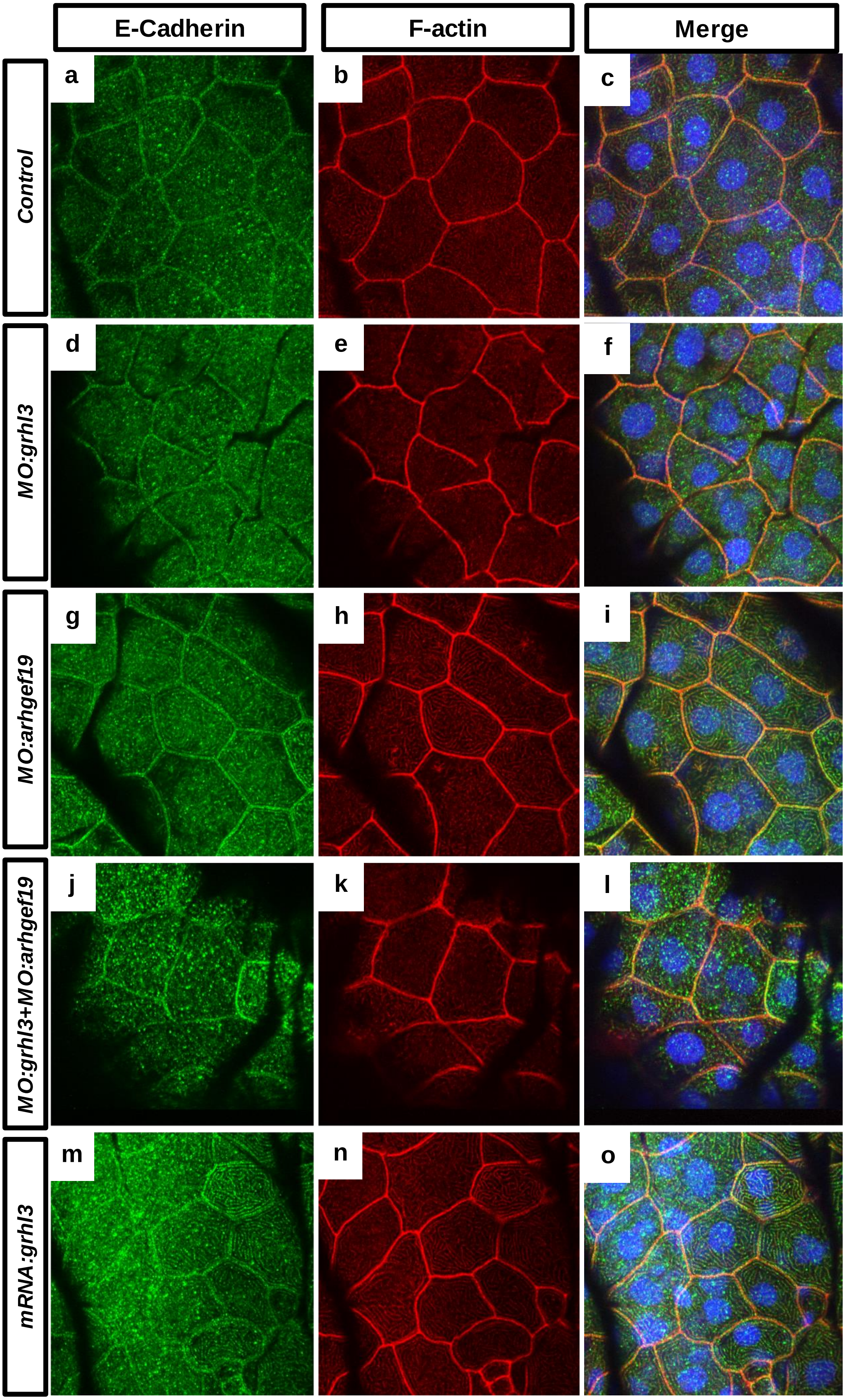

E-Cadherin
F-actin
Merge
Control
MO:grhl3
MO:arhgef19
MO:grhl3+MO:arhgef19
mRNA:grhl3
a
b
c
d
e
f
g
h
i
j
k
l
m
n
o

Fig. S7

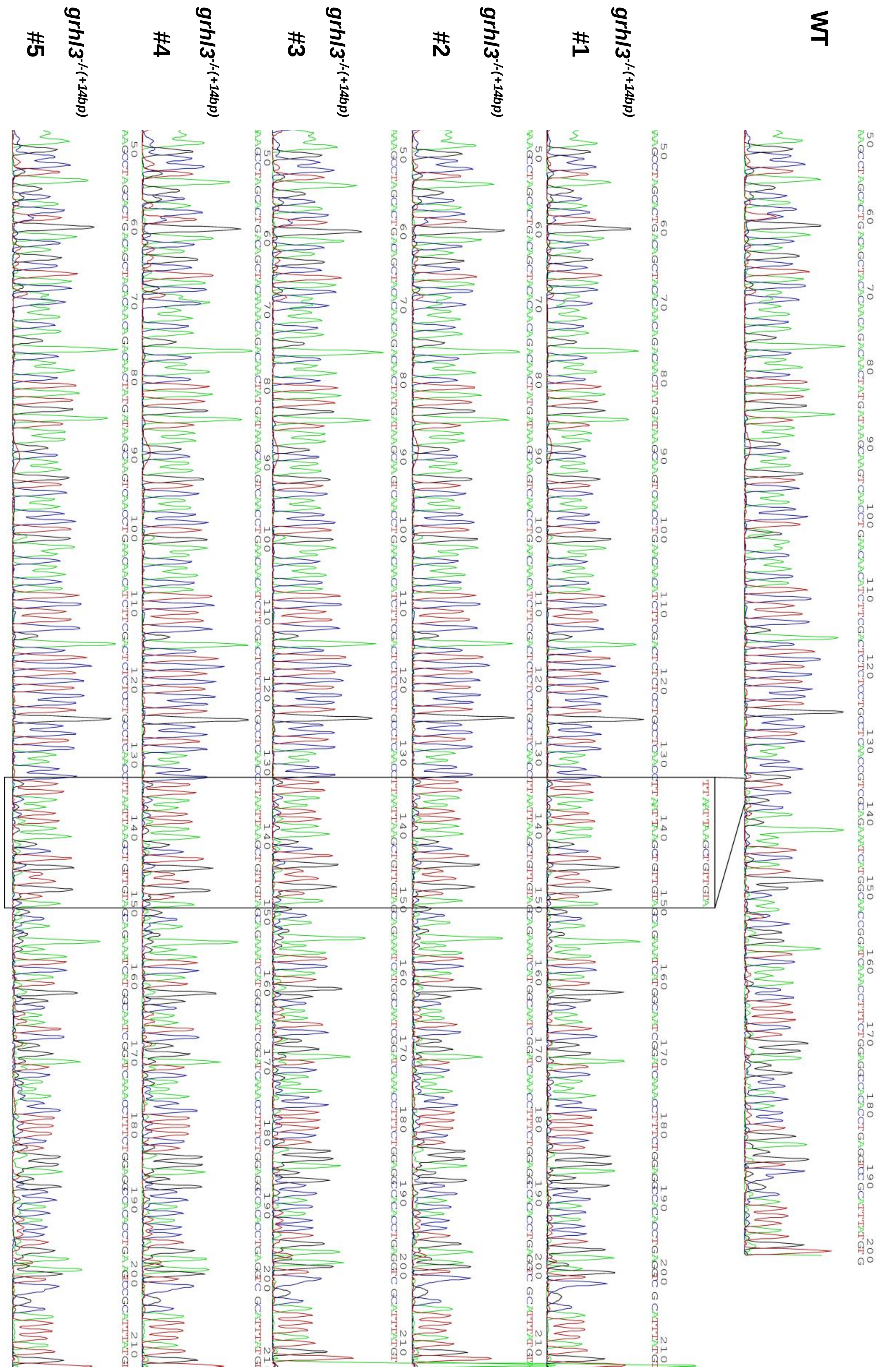
WT
grhl3-/-(+14bp) #1
grhl3-/-(+14bp) #2
grhl3-/-(+14bp) #3
grhl3-/-(+14bp) #4
grhl3-/-(+14bp) #5

Fig. S8

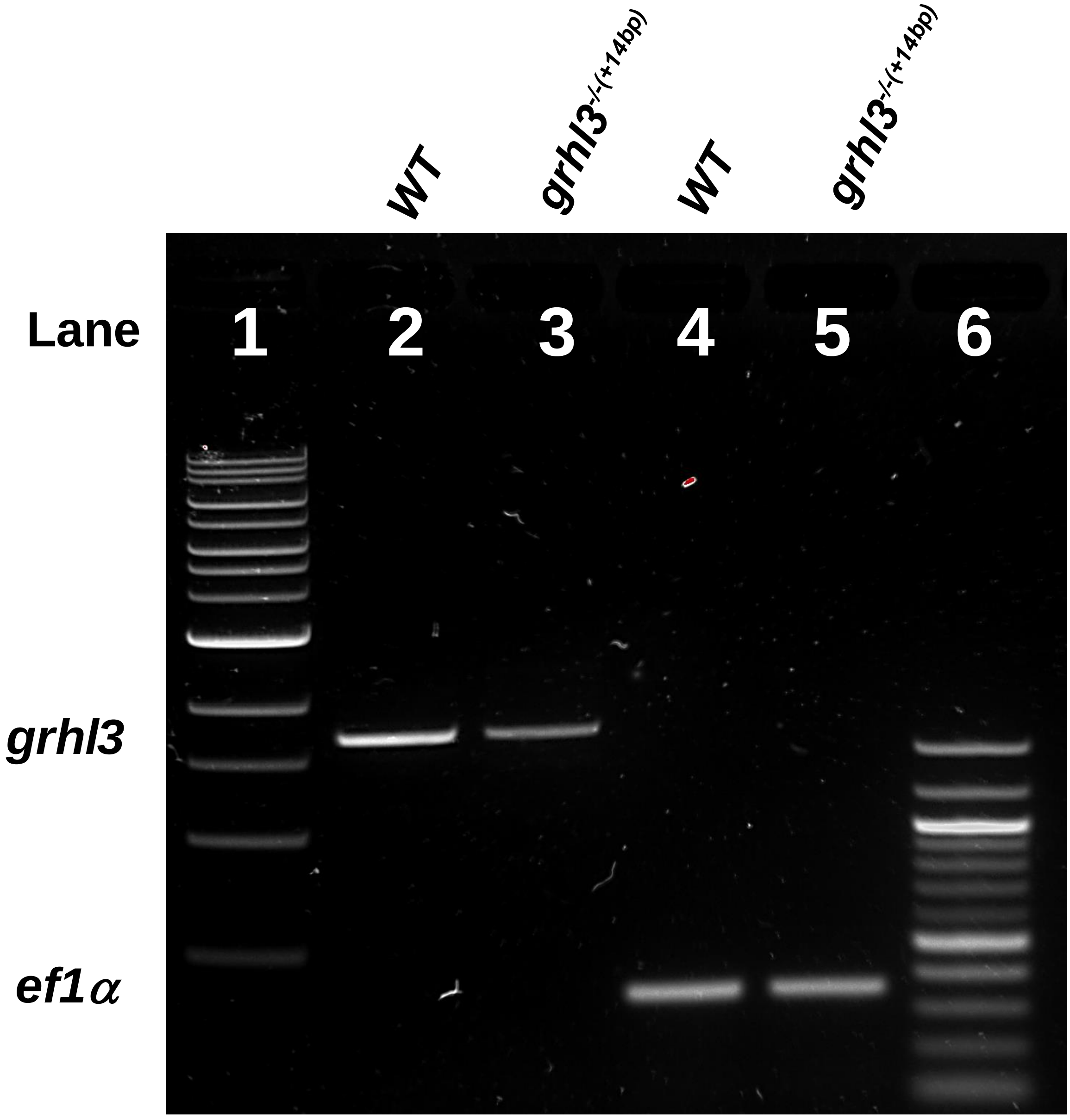

{ } (μM)	**# with**	**% with**
MO:grhl3_ATG block	**MHB defect**	**MHB defect**
500	40/50	80.0%
333	68/125	54.4%
250	60/164	36.6%
200	75/186	40.3%
167	90/260	34.6%
125	181/745	24.3%
MO:grhl3_Splice block		
200	76/145	52.4%
167	17/75	22.7%
125	22/101	21.8%
100	2/16	12.5%

Table S1 – Phenotypic incidence and penetrance following *grhl3* knockdown using ATG and Splice-blocking morpholinos.

{ } (µM; each MO)	**# with MHB defect**	**% with MHB defect**
MO:grhl3+MO:spec1		
167	43/157	27.4%
125	27/172	15.7%
MO:grhl3+MO:arhgef19		
167	22/54	40.7%
125	24/81	29.6%
MO:grhl3+MO:cont		
167	23/125	18.4%
125	5/27	18.5%
MO:spec1+MO:cont		
167	16/196	8.2%
125	4/112	3.6%
MO:arhgef19+MO:cont		
200	2/62	3.2%
125	0/36	0.0%

Table S2 – Phenotypic incidence and penetrance following combinatorial knockdown of *grhl3, spec1* and *arhgef19*.

{ } (μM; each MO)	**# with MHB/CE defects**	**% with MHB/CE defects**
MO:grhl2b+MO:grhl3		
250	58/60	96.7%
200	185/279	66.3%
167	107/262	40.8%
125	171/546	31.3%
MO:grhl2b+MO:cont		
125	4/81	4.9%
100	2/167	1.2%
MO:grhl3+MO:cont		
125	8/54	14.8%
100	9/113	8.0%

Table S3 – Phenotypic incidence and penetrance following combinatorial knockdown of *grhl2b* and *grhl3*.

{ } (ng/µl)	# with	% with	# with	% with	# with	% with	Total #	Total %
zgrhl3 mRNA	FTEA	FTEA	Dorsalisation	Dorsalisation	Twin Axes	Twin Axes	Phenotypes	Phenotypes
320	8/20	40.0%	7/20	35.0%	5/20	25.0%	20/20	100.0%
160	4/10	40.0%	3/10	30.0%	3/10	30.0%	7/10	100.0%
21.3	5/14	35.7%	3/14	21.4%	1/14	7.1%	6/14	64.3%
12.8	36/91	39.6%	18/91	19.8%	19/91	20.9%	55/91	80.2%
10.5	6/32	18.8%	4/32	12.5%	5/32	15.6%	11/32	46.9%
8.5	6/38	15.8%	3/38	7.9%	6/38	15.8%	12/38	39.5%
6.4	16/101	15.8%	10/101	9.9%	8/101	7.9%	24/101	33.7%
5.1	3/31	9.7%	0/31	0.0%	1/31	3.2%	13/31	12.9%
mGrhl3 mRNA								
66	10/43	23.3%	9/43	10.1%	26/43	60.5%	36/43	93.8%
44	6/20	30.0%	3/20	15.0%	6/20	30.0%	12/20	75.0%
33	23/73	31.5%	17/73	10.1%	24/73	32.9%	47/73	74.5%
22	5/41	12.2%	3/41	7.3%	9/41	22.0%	14/41	41.5%
16.5	10/99	10.1%	7/99	7.1%	23/99	23.2%	32/99	40.4%

Table S4 – Quantitation of phenotypes observed following over-expression of *Grhl3* and *grhl3*.

{ } (ng/µl)	# with MHB defect	% with MHB defect	# with partial cyclopia	% with partial cyclopia	# with complete cyclopia	% with complete cyclopia	# with twin axes	% with twin axes	Total # with phenotypes	Total % with phenotypes
grhl2b mRNA										
72	2/34	5.9%	8/34	23.5%	13/34	38.2%	4/34	11.8%	27/34	79.4%
36	1/63	1.6%	8/63	12.7%	22/63	34.9%	2/63	3.2%	33/63	52.4%
7.2	0/6	0.0%	0/6	0.0%	1/6	16.7%	1/6	16.7%	2/6	33.3%
grhl2b-FLAG mRNA										
45	0/37	0.0%	5/37	13.5%	12/37	32.4%	2/37	5.4%	19/37	51.4%
36	0/144	0.0%	14/144	9.7%	26/144	18.1%	0/144	0.0%	40/144	27.8%
mGrhl2 mRNA										
18	0/57	0.0%	7/57	12.3%	9/57	15.8%	3/57	0.0%	19/57	33.3%
9	0/86	0.0%	4/86	4.7%	5/86	5.8%	0/86	0.0%	9/86	10.5%
4.5	0/98	0.0%	4/98	4.1%	0/98	0.0%	0/98	0.0%	4/98	4.1%

Table S5 – Quantitation of phenotypes observed following over-expression of *Grhl2* and *grhl2b*.
